# A kinome-wide RNAi screen identifies ALK as a target to sensitize neuroblastoma cells for HDAC8-inhibitor treatment

**DOI:** 10.1038/s41418-018-0080-0

**Published:** 2018-03-07

**Authors:** Jing Shen, Sara Najafi, Sina Stäble, Johannes Fabian, Emily Koeneke, Fiona R. Kolbinger, Jagoda K. Wrobel, Benjamin Meder, Martin Distel, Tino Heimburg, Wolfgang Sippl, Manfred Jung, Heike Peterziel, Dominique Kranz, Michael Boutros, Frank Westermann, Olaf Witt, Ina Oehme

**Affiliations:** 1 Clinical Cooperation Unit Pediatric Oncology, German Cancer Research Center (DKFZ) and German Cancer Consortium (DKTK), Heidelberg, Germany; 2 Preclinical Program, Hopp Children’s Cancer Center at NCT Heidelberg (KiTZ), Heidelberg, Germany; 3 Present Address: Phenex Pharmaceuticals AG, Heidelberg, Germany; 4 Institute for Cardiomyopathies Heidelberg, Heidelberg University, Heidelberg, Germany; 5 Innovative Cancer Models, St. Anna Children’s Cancer Research Institute, Vienna, Austria; 6 Institute of Pharmacy, Martin-Luther University of Halle-Wittenberg, 06120 Halle/Saale, Germany; 7 Institute of Pharmaceutical Sciences, University of Freiburg, 79104 Freiburg, Germany; 8 Division of Signaling and Functional Genomics, German Cancer Research Center and Heidelberg University, Department for Cell and Molecular Biology, Medical Faculty Mannheim, Heidelberg, Germany; 9 Research Group Neuroblastoma Genomics, German Cancer Research Center, Heidelberg, Germany; 10 Department of Pediatric Oncology, Hematology and Immunology, University of Heidelberg Medical Center, Heidelberg, Germany

## Abstract

The prognosis of advanced stage neuroblastoma patients remains poor and, despite intensive therapy, the 5-year survival rate remains less than 50%. We previously identified histone deacetylase (HDAC) 8 as an indicator of poor clinical outcome and a selective drug target for differentiation therapy in vitro and in vivo. Here, we performed kinome-wide RNAi screening to identify genes that are synthetically lethal with HDAC8 inhibitors. These experiments identified the neuroblastoma predisposition gene *ALK* as a candidate gene. Accordingly, the combination of the ALK/MET inhibitor crizotinib and selective HDAC8 inhibitors (3–6 µM PCI-34051 or 10 µM 20a) efficiently killed neuroblastoma cell lines carrying wildtype *ALK* (SK-N-BE(2)-C, IMR5/75), amplified *ALK* (NB-1), and those carrying the activating *ALK* F1174L mutation (Kelly), and, in cells carrying the activating R1275Q mutation (LAN-5), combination treatment decreased viable cell count. The effective dose of crizotinib in neuroblastoma cell lines ranged from 0.05 µM (*ALK*-amplified) to 0.8 µM (wildtype *ALK*). The combinatorial inhibition of ALK and HDAC8 also decreased tumor growth in an *in vivo* zebrafish xenograft model. Bioinformatic analyses revealed that the mRNA expression level of *HDAC8* was significantly correlated with that of *ALK* in two independent patient cohorts, the Academic Medical Center cohort (*n* = 88) and the German Neuroblastoma Trial cohort (*n* = 649), and co-expression of both target genes identified patients with very poor outcome. Mechanistically, HDAC8 and ALK converge at the level of receptor tyrosine kinase (RTK) signaling and their downstream survival pathways, such as ERK signaling. Combination treatment of HDAC8 inhibitor with crizotinib efficiently blocked the activation of growth receptor survival signaling and shifted the cell cycle arrest and differentiation phenotype toward effective cell death of neuroblastoma cell lines, including sensitization of resistant models, but not of normal cells. These findings reveal combined targeting of ALK and HDAC8 as a novel strategy for the treatment of neuroblastoma.

## Introduction

Neuroblastoma is the most common extracranial solid tumor in children and is derived from precursor cells of the peripheral sympathetic nervous system. The 5-year overall survival probability of high-risk neuroblastoma patients is less than 50% [1]. Moreover, chemotherapy-treated patients struggle with therapy-related immediate and long-term toxicities (reviewed in Brodeur [2]). Thus, more neuroblastoma-specific therapeutic approaches focusing on oncogenic molecular targets are required to improve therapeutic efficacy, reduce toxicity and avoid long-term side effects.

Small molecules that influence gene transcription are also of high interest for the treatment of cancer. One class of drugs in this category are histone deacetylase (HDAC) inhibitors, such as vorinostat (SAHA: suberoylanilide hydroxamic acid), the first clinical HDAC inhibitor approved by the FDA for the treatment of refractory cutaneous T-cell lymphoma [3]. Most inhibitors of HDAC enzymatic activity bind to the highly conserved catalytic domain and hence unselectively inhibit the activity of all zinc-dependent HDAC family members [4]. The enzyme family is grouped into four classes based on their homology to yeast HDACs. In the strict sense, HDACs are more general lysine deacetylase (KDACs), as these enzymes remove acetyl groups from lysine residues of numerous nuclear and cytosolic proteins, affecting gene transcription as well as many cellular pathways [5, 6]. Three of the four classes (class I, II, and IV) have a zinc-dependent catalytic mechanism and constitute the so-called classical HDACs. HDAC family member 8 (HDAC8) together with HDACs 1, 2, and 3 comprise class I [7]. HDAC8 may be an attractive selective target with specific features, as crystal structure analysis revealed a unique second metal binding site in close proximity to the main catalytic domain [8], allowing the design of HDAC8-selective inhibitors. Furthermore, we have previously shown that HDAC8 expression is correlated with advanced tumor stage and poor outcome in neuroblastoma patients [9]. Selective inhibition of HDAC8 slows neuroblastoma growth, induces a more differentiated phenotype and serves as a potent enhancer of retinoic acid-mediated anti-neuroblastoma activity both in vitro and in vivo [10].

RNA interference (RNAi) screens are commonly applied to identify novel limiting factors for drug responsiveness and to unravel targeted combinations of specific drugs to overcome these limitations [11]. Here, we used a kinome-wide RNAi screen to identify new combinations that enhance the sensitivity of neuroblastoma to HDAC8 inhibitors. We identified receptor tyrosine kinases (RTKs), such as anaplastic lymphoma kinase (ALK), as druggable neuroblastoma cell survival activators that can be targeted by treatment with small molecule inhibitors, thus sensitizing neuroblastomas to HDAC8 inhibition.

## Results

### Kinome-wide siRNA screen identifies druggable kinases for the sensitization of neuroblastoma cells to HDAC8 inhibitor treatment

**Fig. 1:**
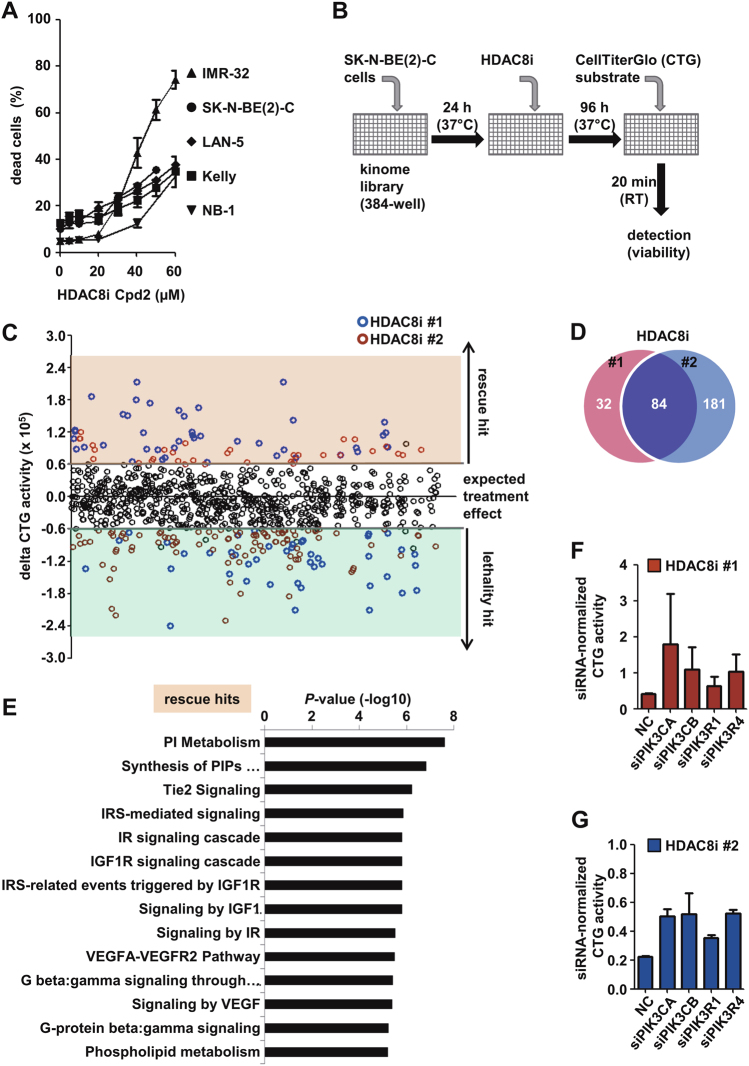
Kinome-wide RNAi screen for the identification of HDAC8 inhibitor co-dependencies. **a** IMR-32, SK-N-BE(2)-C, LAN-5, Kelly and NB-1 cell lines were treated with increasing concentrations of Cpd2 and then monitored for 96 h after treatment for cell death using trypan blue staining (dead cells: trypan blue-positive cells). **b** Schematic of the RNAi screen. **c** Hit plot displaying “rescue hits” and “lethality hits” above or below the expected treatment effect area. **d** Venn diagram showing the overlay of identified hits between both HDAC8 inhibitors. **e** Horizontal bar diagram displaying the *P*-values for significantly enriched pathways (REACTOME; http://www.reactome.org/). **f**, **g** “Rescue hit” examples from the RNAi screen. The bar diagrams display CTG activity upon 96 h of HDAC8i #1 (**f**) and #2 (**g**) treatment normalized for each siRNA pool. **b**–**d**, **f**, **g** HDAC8i: HDAC8 inhibitor; HDAC8i #1: Cpd2 (red), HDAC8i #2: PCI-34051 (blue)

**Fig. 2:**
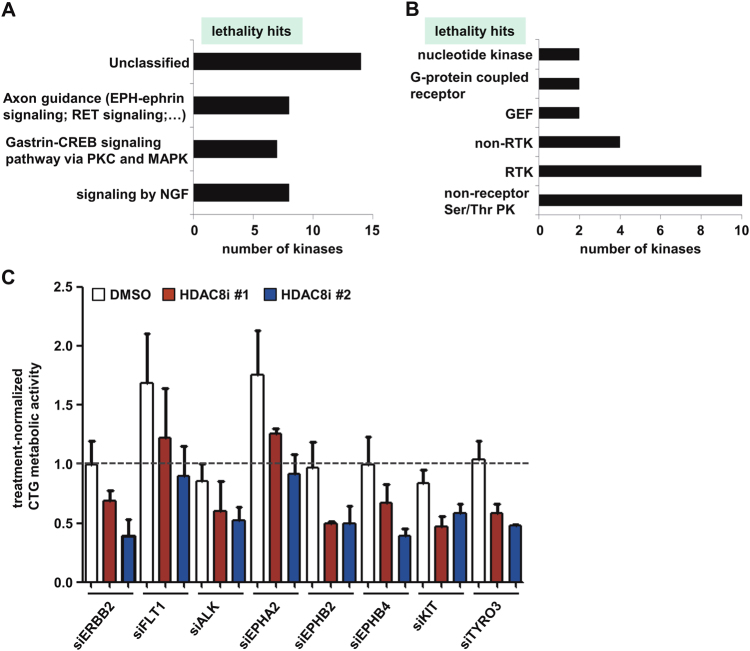
Identification of HDAC8 inhibitor sensitization hits. **a** Horizontal bar diagram displaying significantly enriched signaling pathways for “lethality” hits detected via REACTOME pathway analysis. **b** Horizontal bar diagram displaying the distribution of protein classes for “lethality” hits based on the PANTHER classification system (pthr 24416). Only classes with at least two kinases are shown. PK, protein kinase. RTK, receptor tyrosine kinase. GEF, guanyl-nucleotide exchange factor. **c** “Lethality hit” examples from the RNAi screen. The bar diagram displays CTG activity upon knockdown of the eight RTKs, identified in (**b**), 96 h of HDAC8i treatment. Data are normalized for treatment with each compound

Although HDAC8 inhibitor application significantly slows tumor growth in vivo, the therapeutic effect of treatment with a single HDAC8 inhibitor is not sufficient to induce complete tumor regression, as desired [10]. Cell culture experiments revealed a very strong response in sensitive neuroblastoma cell line IMR-32, whereas other neuroblastoma cell lines investigated responded to HDAC8 inhibition with cell cycle arrest and signs of differentiation rather than cell death (Fig. 1a and [10]). To detect HDAC8 co-dependencies and druggable co-targets, we performed an HDAC8 inhibitor synthetic lethal screen using the siKINOME SMARTpool library (Dharmacon) using short interfering RNA (siRNA) pools consisting of four single siRNAs each, targeting approximately 780 human protein kinases and kinase-associated genes. The SK-N-BE(2)-C cell line was chosen for screening because it exhibits an intermediate response to HDAC8 inhibitor treatment and is one of the most aggressive neuroblastoma cell lines derived from a relapsed *MYCN*-amplified tumor with a *TP53* mutation [12]. Three treatment conditions were applied: solvent and treatment with two structurally different selective HDAC8 inhibitors (Cpd2 [13] and PCI-34051 [14]). Comparison of duplicate experiments revealed high reproducibility of the screen (Supplementary Figure 1A). The screen was optimized to detect sensitizing (“lethal”) and inhibitory (“rescue”) effects by incubating cells with the IC50 concentration of HDAC8 inhibitors (40 µM Cpd2 and 4 µM PCI-34051). After 96 h, neuroblastoma cell viability was assessed by Cell Titer Glo (CTG) assays (Fig. 1b). Data were normalized to the respective treatment with dimethyl sulfoxide (DMSO) or HDAC8i (Supplementary Figure 1B–D). A hit was defined as HDAC8i-normalized treatment minus DMSO-normalized treatment >60,000 RLU (=rescue hit; orange shading) or <−60,000 RLU (=lethality hit; green shading) (Fig. 1c; Supplementary Table 1). This cut-off separates the candidates of interest (blue HDAC8i #1, red HDAC8i #2) from the expected treatment effect (black circles of all treatments). Finally, hits were defined as only those candidates whose effects were reproducible for both replicates and both HDAC8 inhibitors. In total, the screen identified 84 common hits (Fig. 1d): 41 rescue hits, and 43 lethality hits (Supplementary Table 1). Analysis of the “rescue hit list” by gene ontology enrichment analysis with GOrilla revealed the overrepresentation of phosphatidylinositol kinase and phosphatidylinositol bisphosphate kinase activity (Supplementary Figure 1E), which was confirmed by pathway analysis with REACTOME (cut-off *p* < 0.00001; Fig. 1e). This suggests that PI3K pathway inactivation abolishes the anti-neuroblastoma effect of the HDAC8 inhibitor, exemplarily shown for knockdown of PIK3CA, PIK3CB, PIK3R1 and PIK3R4 in Fig. 1f, g. REACTOME pathway analysis of the “lethality hit list” revealed axon guidance (e.g., RET and Ephrin signaling), CREB-PKC/MAPK signaling and NGF signaling to be significantly enriched (Fig. 2a). A protein class analysis (PANTHER) [15] indicated an enrichment of non-receptor serine-threonine kinases and RTKs among the lethality hits (Fig. 2b). Figure 2c shows the treatment sensitizing effect of knockdown of the eight RTKs (ERBB2, FLT1, ALK, EPHA2, EPHB2, EPHB4, KIT, and TYRO3). Besides HDAC8 inhibitor-specific hits, general toxicity hits of neuroblastoma, such as the known effectors of cell viability, PLK1 [16] and WEE1 [17, 18], were identified in the screen and are listed in Supplementary Table 2. Overall, the kinome-wide RNAi screen identified the PI3K pathway to be involved in HDAC8 inhibitor-mediated anti-neuroblastoma effects and identified additional druggable RTKs, such as ERBB2 and ALK, as targets to sensitize neuroblastoma to HDAC8 inhibitor treatment.

### Combined ALK and HDAC8 inhibitor treatment on ALK wild-type neuroblastoma cells

We selected one RTK, ALK, for subsequent studies, as the gene encoding for ALK was identified as a major familial neuroblastoma predisposition gene [19] and can be targeted by US Food and Drug Administration (FDA)-approved drugs. The ALK/MET/ROS1 inhibitor crizotinib [20] is approved for the treatment of non-small cell lung carcinoma (NSCLC) with ALK translocations [21–23] and is also being tested in neuroblastoma (clinical trial: NCT00939770) [24]. Furthermore, the ALK inhibitor LDK378 is being investigated in clinical trials for the treatment of pediatric malignancies with a genetic alteration of ALK (NCT01742286). Schulte et al. [25] recently demonstrated high ALK expression in primary neuroblastoma as a determining factor of an unfavorable phenotype.

**Fig. 3:**
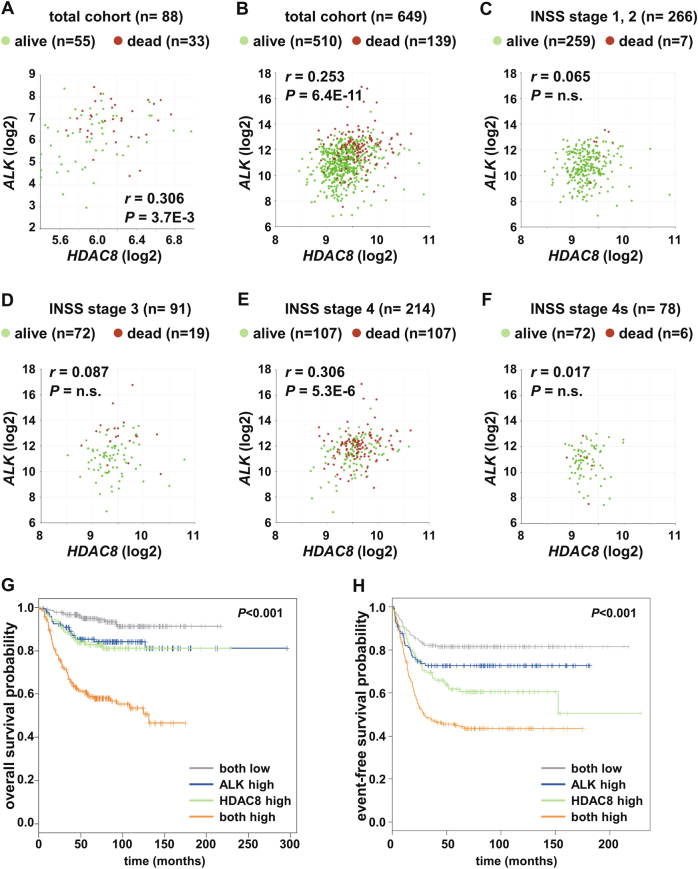
Correlated ALK and HDAC8 expression in INSS stage 4 patients. (**a**–**f**) Scatter plots are shown for the expression of HDAC8 and ALK in neuroblastoma patient cohorts; green dots, alive; red dots, dead. The R2 platform was used for the calculation, and the AMC cohort “Neuroblastoma public—Versteeg −88” (MAS5.0–u133p2) (**a**) as well as the Cologne cohort “Neuroblastoma—Kocak −649” (custom.ag44kcwolf) (**b**–**h**) was the source of the data. The following probe sets were used: ALK 208211_s_at and HDAC8 223345_at (AMC) and ALK UKv4_A_23_P324304 and HDAC8 UKv4_A_P254965 (Kocak). INSS: International Neuroblastoma Staging System. **a** Whole cohort, Versteeg. **b** Whole cohort, Kocak. **c** INSS stage 1, 2. **d** iNSS stage 3. **e** INSS stage 4. **f** INSS stage 4 s. **g** Kaplan–Meier curves are shown for the overall survival of neuroblastoma patients with tumors expressing low levels of both HDAC8 and ALK (both low, gray, *n* = 205), low levels of HDAC8 and high levels of ALK (ALK high, blue, *n* = 121), high levels of HDAC8 and low levels of ALK (HDAC8 high, green, *n* = 122), or high levels of both HDAC8 and ALK (both high, orange, *n* = 201). (**h**) Kaplan-Meier curves are shown for the event-free survival of neuroblastoma patients with tumors expressing low levels of both HDAC8 and ALK (both low, gray, *n* = 201), low levels of HDAC8 and high levels of ALK (ALK high, blue, *n* = 112), high levels of HDAC8 and low levels of ALK (HDAC8 high, green, *n* = 119), or high levels of both HDAC8 and ALK (both high, orange, *n* = 196). **g**, **h** The *p*-value was determined using a log-rank test. Median expression was used as the cut-off

ALK expression studies using two publicly available neuroblastoma cohorts in the R2 database [26], the Academic Medical Center (AMC) cohort with 88 patient samples and the large cohort of neuroblastoma cases (*n* = 649) from the German Neuroblastoma Trial [27], revealed a strong correlation with *HDAC8* expression (Fig. 3a, b). When the large cohort was separated by stage (Fig. 3c–f), a strong, significant correlation was only found in International Neuroblastoma Staging System (INSS) stage 4 patients (Fig. 3e). Accordingly, the co-expression of both genes, *ALK* and *HDAC8*, was correlated with poor survival of neuroblastoma patients in both cohorts, with long-term overall and event-free survival below 50% (Table 1; Fig. 3g, h). Cox regression analysis identified that, even after accounting for the effects of stage, high HDAC8 co-expressed with ALK is a significant risk factor for poor outcomes in neuroblastoma patients (Supplementary Table 3), further supporting the investigation of combining an HDAC8 inhibitor with an ALK inhibitor for neuroblastoma treatment.

**Table 1:** Ten year Kaplan–Meier survival probability based on *HDAC8* and *ALK* expression

		Overall survival (95% CI)	Event-free survival (95% CI)
*HDAC8* low/*ALK* low	German NB Trial	0.911 (0.866–0.959)	0.815 (0.763–0.871)
	AMC Cohort	0.821 (0.691–0.976)	0.75 (0.606–0.929)
*HDAC8* high/*ALK* high	German NB Trial	0.534 (0.458–0.623)	0.434 (0.369–0.512)
	AMC Cohort	0.481 (0.326–0.712)	0.418 (0.263–0.663)

*CI* confidence interval, *German NB Trial* German Neuroblastoma Trial; *AMC* Academic Medical Center (University of Amsterdam)

Survival rates calculated using Kaplan–Meier estimator. “Low” expression indicates an expression level below the median expression for that gene. “High” expression reflects expression above the median

**Fig. 4:**
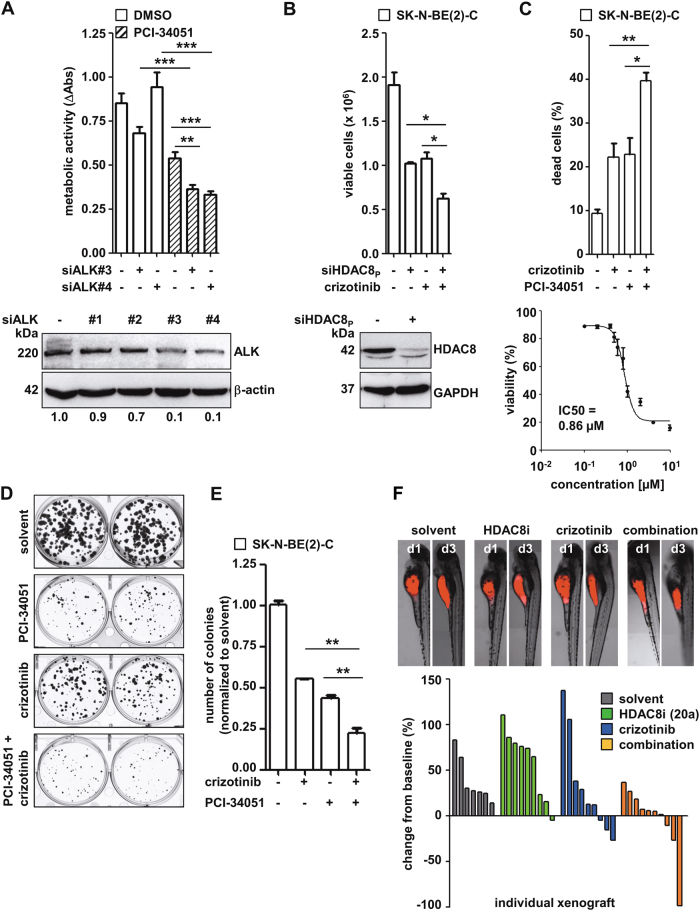
Validation of ALK as a target for HDAC8 inhibitor sensitization. **a** Upper: The SK-N-BE(2)-C cell line was transfected with ALK siRNA #3 and #4 or negative control siRNA (NC) and 48 h after transfection treated with 6 μM PCI-34051 for 96 h. Metabolic activity was measured by an WST-8 assay and is shown relative to untransfected, untreated cells. Lower: Western blot displaying ALK levels 72 h after transfection with siRNAs #1–4. Actin served as a loading control. **b** Upper: The SK-N-BE(2)-C cell line was transfected with HDAC8 siRNA pool (siHDAC8_P_: siRNA #1 and #2) or negative control siRNA (minus sign) and 24 h later was treated with crizotinib (0.8 µM, 96 h). The number of viable cells was assessed by automated cell counting and trypan blue exclusion. Lower panel: Western blot displaying HDAC8 levels 72 h after transfection. GAPDH served as a loading control. **c** Upper panel: SK-N-BE(2)-C cells were treated with crizotinib (0.8 µM) alone or in combination with PCI-34051 (6 µM) and then monitored for 96 h after treatment for cell death using trypan blue staining (dead cells: trypan blue-positive cells). Lower: SK-N-BE(2)-C cells were treated 24 h post-seeding with various concentrations of crizotinib for 96 h. IC50 values were calculated using GraphPad Prism. Viability (%) was assessed by automated cell counting and trypan blue exclusion. **d** SK-N-BE(2)-C cells (800 cells/well) were treated with crizotinib (0.8 µM) alone or in combination with PCI-34051 (6 µM), and colonies were stained after 10 days. **e** Bar diagram displaying the quantified colonies. **f** Upper: representative pictures of zebrafish larvae with tumors formed by fluorescently-labeled SK-N-BE(2)-C cells that were injected into the yolk sac. Images are shown for each treatment condition on day 1 post-implantation (d1; start of treatment) and day 3 post-implantation (d3; end of experiment). Lower: waterfall plot displaying the change in tumor size (%) from baseline (day 1 = start of treatment) to day 3 after implantation for each individual xenograft. Zebrafish embryos were treated with a ten-fold higher concentrations as substances are applied to the surrounding water and, as the estimated extent of compound absorption by the zebrafish larvae is one-tenth to one-twentieth of the cell culture treatment concentration. Solvent: DMSO, gray; HDAC8i: 100 µM 20a, green; crizotinib: 8 µM, blue; combination: 100 µM 20a and 8 µM crizotinib (orange). **a**–**e** Means from at least three independent experiments are shown, and the error bars represent SEM. *p < 0.05; **p < 0.01; ***p < 0.001

To further validate ALK as a suitable target for the sensitization of neuroblastoma cells to HDAC8 inhibitor treatment, SK-N-BE(2)-C cells were transfected with the two most effective single ALK siRNAs and treated with the HDAC8 inhibitor PCI-34051 [14] (Fig. 4a), and *vice versa*, SK-N-BE(2)-C cells transfected with an siRNA pool against HDAC8 (Fig. 4b) were treated with crizotinib. In both conditions, combination treatment significantly reduced cellular viability, which served as an indicator of anti-tumoral effects. Regarding the reduction in cell viability, the IC50 of crizotinib was determined to be 0.86 µM in SK-N-BE(2)-C cells (Fig. 4c). The peak plasma concentration of crizotinib has been estimated to be approximately 1.4 µM in patients [24]. The combination of 0.8 µM crizotinib with 6 µM PCI-34051 increased the amount of dead SK-N-BE(2)-C cells from approximately 20 up to 40% (Fig. 4c). We next performed colony formation assays to further evaluate the potential therapeutic effects of the combined treatment against neuroblastoma cells. Significantly fewer colonies were formed in the combination treatment groups compared to the single treatment group (Fig. 4d, e). In line with this result, the combination of an HDAC8 inhibitor and crizotinib impaired tumor growth in the SK-N-BE(2)-C zebrafish xenograft in vivo model (Fig. 4f, Supplementary Figure 2A). Engraftment of human tumor cells into zebrafish, which lack an adaptive immune system in the first month of life, is an effective method for early preclinical drug screening [28]. The transparency of zebrafish embryos allows tracking of fluorescently-labeled SK-N-BE(2)-C cells and monitoring of tumor formation and progression using confocal microscopy. The efficacy of HDAC8 inhibition, crizotinib and the combination was assessed by evaluating tumor volume change, quantified via semi-automated image analysis, from day one (start of treatment) to day three (end of experiment) post-implantation_._ Here, we used the novel compound 20a, a hydroxamate-based inhibitor selective for HDAC8, not targeting HDAC1–3 [29], since PCI-34051 was toxic to zebrafish embryos (Supplementary Figure 2B). Overall, our results imply sensitizing effects of the combinatorial treatment of crizotinib with HDAC8 inhibition in SK-N-BE(2)-C cells.

### Dual targeting of ALK and HDAC8 also eliminates neuroblastoma cells harboring ALK-activating genetic aberrations

**Fig. 5:**
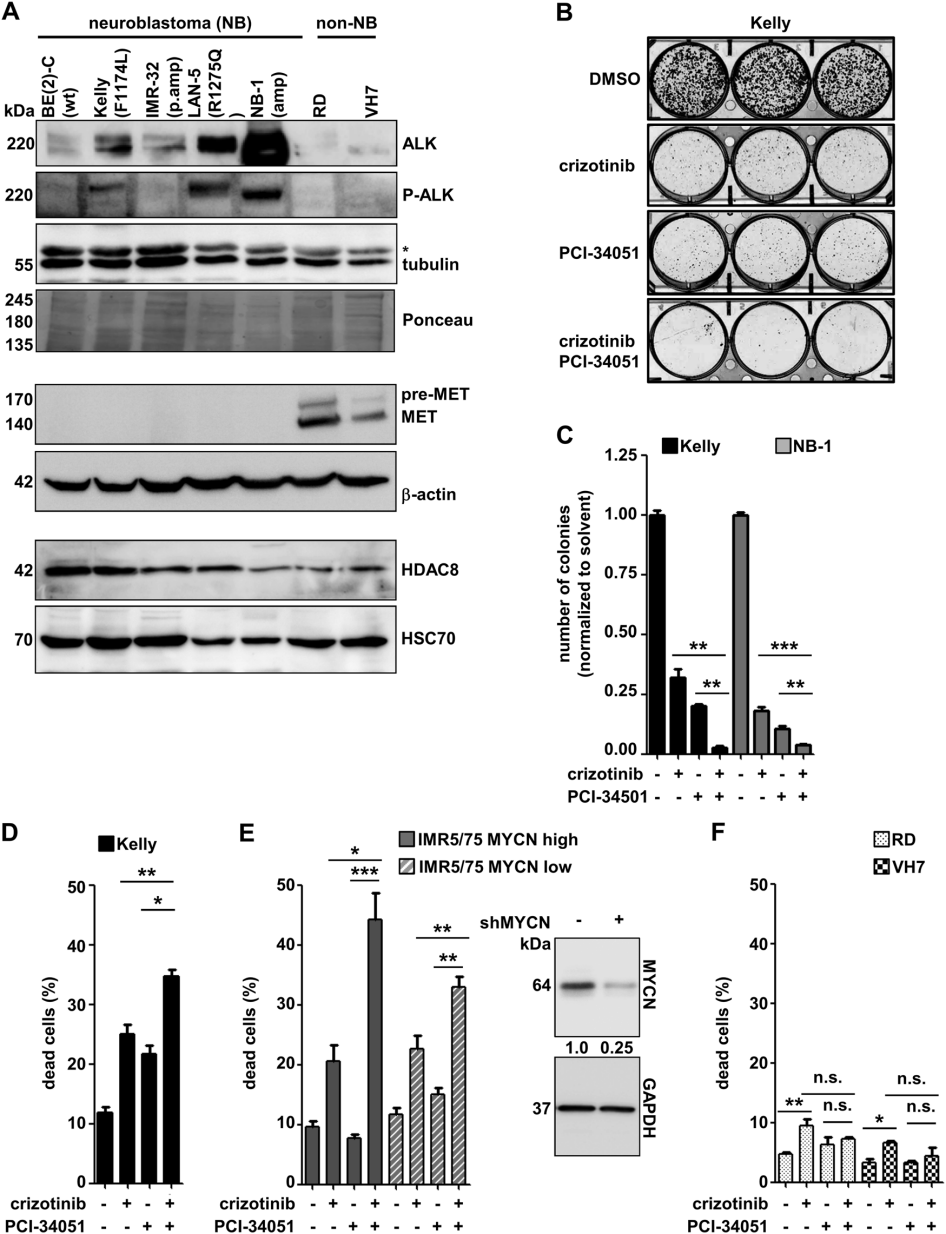
Dual targeting of ALK and HDAC8 efficiently targets neuroblastoma cell lines. **a** Expression of ALK, P-ALK, MET and HDAC8 protein levels in a panel of neuroblastoma (NB) and non-neuroblastoma cell lines. Tubulin, actin and HSC70 as well as Ponceau staining of the membrane served as a loading control. *Unspecific band (**b**) Kelly cells (5000 cells/well) were treated with crizotinib (0.8 µM) alone or in combination with PCI-34051 (6 µM), and colonies were stained after 10 days. **c** Bar diagram displaying the quantification of colonies upon treatment of Kelly and NB-1 cell lines with crizotinib (0.8 µM for Kelly, 0.05 µM for NB-1) or PCI-34051 (6 µM for Kelly, 4 µM For NB-1) alone or in combination. **d** Kelly cells were treated with crizotinib (0.8 µM) alone or in combination with PCI-34051 (6 µM) and then monitored for 96 h after treatment for cell death using trypan blue staining (dead cells: trypan blue-positive cells). **e** IMR5/75 cells containing the tetracycline-inducible system for shRNA-mediated knockdown of MYCN were treated with crizotinib (0.4 µM) alone or in combination with PCI-34051 (3 µM) and then monitored for 96 h after treatment for cell death using trypan blue staining (dead cells: trypan blue-positive cells). Cells were treated with 1 μg/ml doxycycline (*shMYCN*) or solvent control (*off*) 2 weeks prior to compound treatment. Right: Expression of MYCN protein upon induction of knockdown with doxycycline (+) for two weeks. GAPDH served as a loading control. Numbers indicate MYCN expression normalized to GAPDH expression. **f** Non-neuroblastoma cell lines RD and VH7 were treated with crizotinib (0.8 µM) alone or in combination with PCI-34051 (6 µM) and then monitored for 96 h after treatment for cell death using trypan blue staining (dead cells: trypan blue-positive cells). **c**–**f** Means from at least three independent experiments are shown, and the error bars represent SEM. *p < 0.05; **p < 0.01; ***p < 0.001

To expand the panel of investigated cell lines, we determined the ALK expression status of five neuroblastoma cell lines, a cell line from a non-neuroblastoma pediatric embryonal tumor (RD, rhabdomyosarcoma) [30], and a non-malignant but proliferative fibroblast line from an infant donor (VH7) [10]. All neuroblastoma cell lines expressed ALK, with strong expression in LAN-5 cells and amplification in NB-1 cells, whereas the other target of crizotinib, c-MET, was not expressed. However, both non-neuroblastoma control lines (RD and VH7) expressed c-MET while ALK expression was not detectable (Fig. 5a). ALK phosphorylation was detected in Kelly, LAN-5, and NB-1 cells and to a much weaker extent in SK-N-BE(2)-C cells (Fig. 5a). As Kelly cells harbor the constitutively active F1174L ALK mutation, which confers primary resistance to the ALK inhibitor crizotinib [31], we tested our treatment combination in these cells and in NB-1 cells harboring *ALK* and *MYCN* amplification [32]. In colony formation assays, combined treatment of cells with PCI-34051 and crizotinib significantly impaired the ability of both cell lines to form colonies (Fig. 5b, c). The combined treatment of Kelly cells with PCI-34051 and crizotinib enhanced cell death to approximately 35% (Fig. 5d). Significantly higher caspase-3 (DEVDase) activity was observed in the combination treatment group compared to the single treatments in Kelly (ALK F1174L) and NB-1 (ALK-amplified) cells (Supplementary Figure 3A), and the proportion of cells in the subG1 area of the cell cycle was significantly enriched in the combination treatment group (Supplementary Figure 3B). The application of a pan-caspase inhibitor (zVAD.fmk) significantly reduced the amount of dead cells in the combination-treated group (Supplementary Figure 3C), demonstrating that the combination treatment triggers caspase-mediated programmed cell death.

To ensure target specificity, we exchanged crizotinib for LDK378, and PCI-34051 for the novel HDAC8-selective compound 20a [29], and similar results were obtained (Supplementary Figure 3D–F). Since the R1275Q *ALK* mutation occurs frequently in neuroblastoma, we also tested combinatorial inhibition in LAN-5 cells. Significantly fewer viable cells remained after treatment with the combination, however, treatment with crizotinib alone was already quite effective in these cells (Supplementary Figure 3G).

To address the role of the neuroblastoma-specific oncogene *MYCN*, which cooperates with activated ALK during neuroblastoma pathogenesis [33, 34], the inducible IMR5/75 (ALK-amplified) shMYCN knockdown system was utilized [35]. Cells in both conditions, *shMYCN off* (high MYCN) and *shMYCN on* (low MYCN), were treated with the HDAC8 inhibitor, crizotinib or the combination of both. The combinatorial treatment with both inhibitors resulted in increased cell death in IMR5/75 cells. This sensitization effect was weaker with the knockdown of *MYCN* (Fig. 5e), suggesting a sensitizing function of *MYCN* amplification for the dual targeting approach. Of note, though the average cell death rate in non-neuroblastoma and non-ALK expressing cell lines (RD and VH7) was below 10% for all treatment conditions (Fig. 5f), crizotinib treatment increased the amount of dead cells, presumably by interacting with c-MET (Fig. 5a). Altogether, simultaneous inhibition of ALK and HDAC8 eliminates ALK wild-type, constitutively active (F1174L-mutated and ALK-amplified), and *MYCN*-amplified neuroblastoma cell lines, but not other malignant and non-transformed cell lines, and *MYCN* amplification sensitizes neuroblastoma cells to the combination treatment.

### Identification of RTK-ERK signaling pathways in the sensitization to HDAC8 targeting

To mechanistically understand the interplay of HDAC8 inhibition and crizotinib, we next investigated the treatment effects on ALK activation and postulated downstream pathways (STAT3, PI3K-AKT and MAPK-ERK) [31, 36]. The activation of ALK by Y1604 phosphorylation, was not affected by HDAC8 inhibitor treatment (Supplementary Figure 4). To achieve comparable levels of ALK inhibition, SK-N-BE(2)-C cells were treated with 0.6 µM and NB-1 cells with 0.05 µM crizotinib (Supplementary Figure 4).

**Fig. 6:**
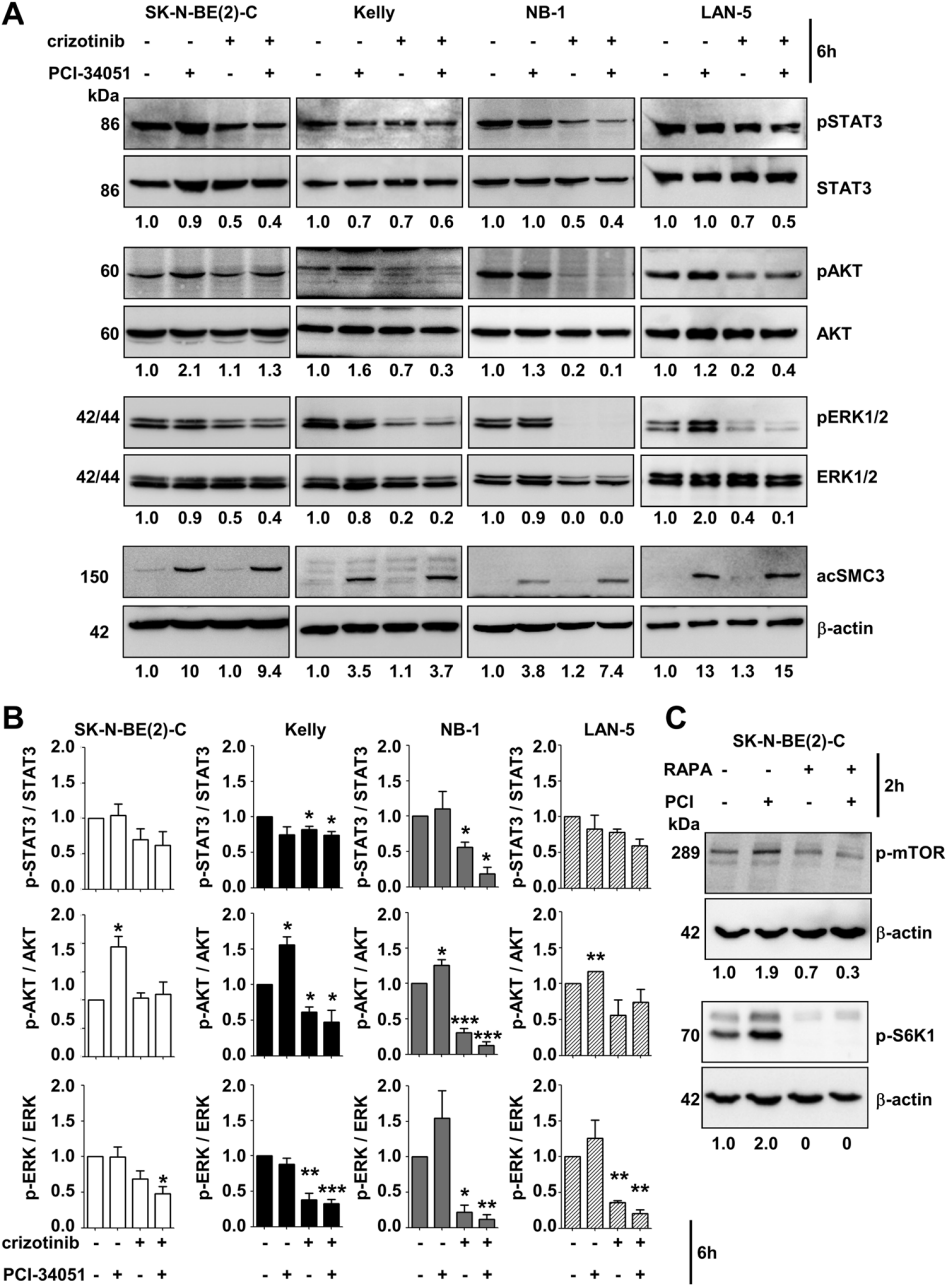
HDAC8 and ALK converge at the level of ALK downstream targets. **a** Detection of phosphorylation levels of STAT3, AKT and ERK1/2 in neuroblastoma cells treated for 6 h with crizotinib (0.6 µM SK-N-BE(2)-C, Kelly and LAN-5, 0.05 µM NB-1) alone or in combination with PCI-34051 (6 µM SK-N-BE(2)-C, Kelly, NB-1 and 3 µM LAN-5). Numbers in the upper row indicate pSTAT3 levels normalized to STAT3 expression, numbers in the middle row indicate pAKT levels normalized to AKT expression, and numbers in the lower row indicate pERK1/2 levels normalized to ERK1/2 expression. Lower: western blot analysis showing acetylation levels of HDAC8 substrate SMC3 in neuroblastoma cells. Actin served as a loading control. **b** The bar diagrams display the quantification of at least three independent experiments from (**a**). Means from at least three independent experiments are shown, and the error bars represent SEM. *p < 0.05; **p < 0.01; ***p < 0.001. A one-sample *t*-test was used to test whether means were significantly different from a hypothetical value (1.0). **c** Detection of phosphorylation levels of mTOR and S6K1 in SK-N-BE(2)-C cells treated for 2 h with PCI-34051 (PCI, 6 µM) alone or in combination with rapamycin (RAPA, 100 nM). Actin served as a loading control

Single treatment of SK-N-BE(2)-C (ALK-wt), Kelly (F1174L), NB-1 (ALK-amp) and LAN-5 (R1275Q) cells with the HDAC8 inhibitor PCI-34051 had no effect on Y705 phosphorylation of STAT3, and had no significant effect on ERK1/2 phosphorylation, but slightly enhanced Y473 phosphorylation of AKT (Fig. 6a, b). In contrast, crizotinib treatment of SK-N-BE(2)-C, Kelly, NB-1 and LAN-5 cells inhibited phosphorylation of STAT3 (Kelly, NB-1) and, to a much higher degree, phosphorylation of ERK1/2, with the strongest inhibitory effect observed in NB-1 cells. The combination of both compounds reversed the HDAC8 inhibitor-mediated effects on AKT phosphorylation and abolished ERK1/2 phosphorylation in all four cell lines with complete abrogation of ERK signaling in NB-1 cells (Fig. 6a, b). The phosphorylation of AKT by HDAC8 inhibitor treatment is in line with the results of the initial RNAi screen, which indicated that the PI3K pathway mediates HDAC8 inhibitor anti-neuroblastoma effects. This PI3K pathway activation is also reflected by enhanced phosphorylation of the AKT downstream target mTOR and phosphorylation of the mTOR substrate S6K (Fig. 6c). Overall, these results confirm the relevance of the PI3K-AKT-mTOR pathway in HDAC8 inhibitor-mediated effects, and ALK inhibition by crizotinib abolished the effect of the HDAC8 inhibitor on AKT. However, inhibition of ALK by crizotinib affects the ERK pathway, and treatment with the HDAC8 inhibitor potentiates inhibition of the ERK pathway.

### Inhibition of RTK-mediated signaling shifts the HDAC8 inhibitor-mediated phenotype from differentiation to cell death

**Fig. 7:**
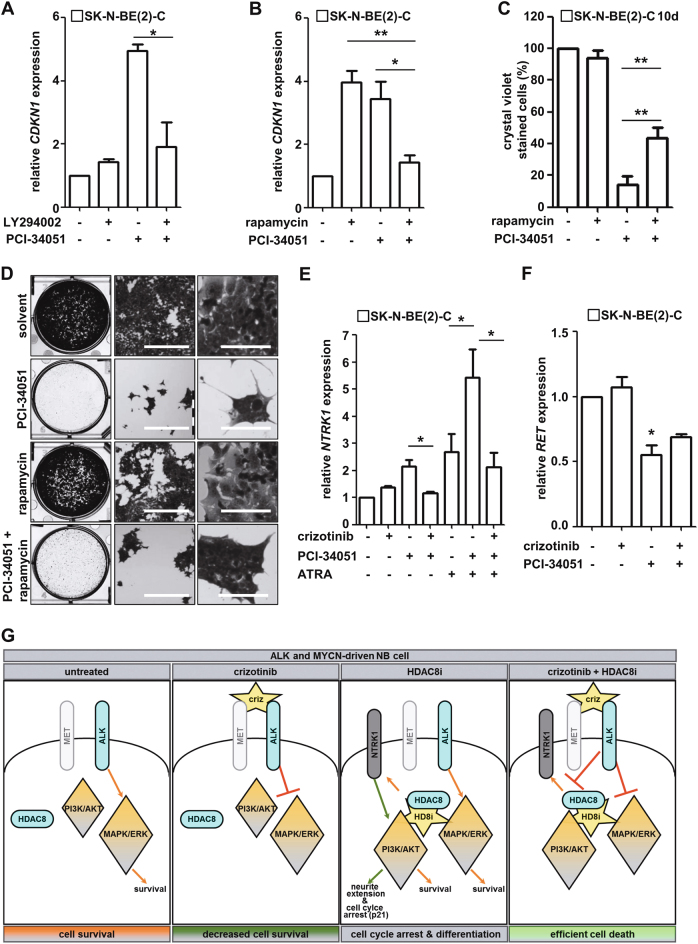
RTK inhibition shifts HDAC8 inhibitor phenotype from differentiation to cell death. **a** SK-N-BE(2)-C cells were treated with LY294002 (10 µM) alone or in combination with PCI-34051 (6 µM), and RNA was isolated 72 h after treatment for real-time PCR analysis of *CDKN1* (p21) expression. **b** SK-N-BE(2)-C cells were treated with rapamycin (100 nM) alone or in combination with PCI-34051 (6 µM), and RNA was isolated 72 h after treatment for real-time PCR analysis of *CDKN1* (p21) expression. **c** The bar diagram displays the ImageJ-based quantification of crystal violet-stained SK-N-BE(2)-C cells 10 days after treatment with rapamycin (100 nM) alone or in combination with PCI-34051 (6 µM). Data were normalized to solvent (DMSO)-treated cells. **d** Representative pictures of crystal violet-stained SK-N-BE(2)-C cells 10 days after treatment with rapamycin (100 nM) alone or in combination with PCI-34051 (6 µM). Scale bars, middle column: 500 µm; scale bars, right column: 100 µm. **e** SK-N-BE(2)-C cells were treated with crizotinib (0.8 µM) alone or in combination with PCI-34051 (6 µM). Where indicated, treatment was additionally combined with ATRA (10 µM). RNA was isolated 72 h after treatment for real-time PCR analysis of *NTRK1* expression. **f** SK-N-BE(2)-C cells were treated with crizotinib (0.8 µM) alone or in combination with PCI-34051 (6 µM), and RNA was isolated 72 h after treatment for real-time PCR analysis of *RET* expression. **g** Model of the lethal interactions of crizotinib with HDAC8 inhibitors. The specific effects of crizotinib on ALK downstream pathways (mainly via ERK inhibition) shift the rather mild HDAC8 inhibitor phenotype, characterized by cell cycle arrest and early signs of differentiation (neurite extension), toward effective neuroblastoma cell death. HD8i = HDAC8i, HDAC8 inhibitor; criz, crizotinib. **a**–**c**, **e**, **f** Means from at least three independent experiments are shown, and the error bars represent SEM. *p < 0.05; **p < 0.01; ***p < 0.001

To further characterize the role of PI3K pathway activity in HDAC8 inhibitor-mediated effects, we treated SK-N-BE(2)-C cells with PCI-34051, the PI3K inhibitor LY294002, the mTOR inhibitor rapamycin or the dual PI3K/mTOR inhibitor BEZ235. Co-treatment blocked the HDAC8 inhibitor-induced *CDKN1* mRNA upregulation (Fig. 7a, b; Supplementary Figure 5A–C), increased the amount of cells surviving long-term HDAC8 inhibitor treatment (Fig. 7c) and reduced the outgrowth of neurofilament-positive structures (Fig. 7d; Supplementary Figure 5D). This indicates that the PI3K-AKT-mTOR axis is relevant for the differentiating phenotype induced by HDAC8 inhibitor treatment of SK-N-BE(2)-C cells, rendering the combination of PI3K/mTOR inhibitors with HDAC8 inhibitors counterproductive. As recent studies showed that HDAC inhibitors act in cooperation with PI3K and mTOR inhibitors to inhibit tumor growth in MYC-driven tumors [37, 38], we tested the HDAC class I inhibitor entinostat (MS-275) at a concentration ineffective for HDAC8 (500 nM) [39] and combined it with the PI3K or mTOR inhibitor. Indeed, both LY294002 and rapamycin significantly enhanced entinostat-mediated effects on cell viability (Supplementary Figure 5E, F), suggesting that HDAC1–3 inhibitors, but not HDAC8 inhibitors, cooperate with PI3K/mTOR inhibitors in our neuroblastoma MYCN-driven model.

As ALK inhibition by crizotinib abolished the effect of the HDAC8 inhibitor on AKT but strongly inhibited ERK, which resulted in enhanced cell death, we hypothesized that RTK inhibition shifts the HDAC8 inhibitor-mediated differentiation phenotype toward neuroblastoma cell death. One characteristic marker of the HDAC8 inhibitor phenotype is up-regulation of *NTRK1* [9], especially upon co-treatment with the differentiation-inducing agent retinoic acid (ATRA) [10]. The combined treatment of SK-N-BE(2)-C cells with PCI-34051 and crizotinib not only blocked HDAC8 inhibitor-induced upregulation of *NTRK1* but also diminished the powerful effect of the PCI-34051/ATRA combination (Fig. 7e). Finally, we asked whether there was an overlap between the published ALK inhibitor gene expression signature [40] and HDAC8 inhibitor-mediated gene expression effects. A subset of ALK signature genes was affected by HDAC8 inhibitor treatment. Seven genes were regulated in the opposite direction (e.g. MAPK negative feedback regulator *SPRY4*) and two genes (*RET*, *VIP*) were regulated in the same direction (Supplementary Figure 5G, Supplementary Table 4). The proto-oncogene and neuronal marker RET has been proposed as a drug target in the context of aberrant ALK activation and its overexpression is driven by mutant ALK [40]. Realtime PCR analysis confirmed downregulation of RET in ALK wildtype SK-N-BE(2)-C cells upon treatment with PCI-34051 (Fig. 7f). In summary, we conclude that targeting ALK diminishes HDAC8 inhibitor treatment-mediated neuroblastoma cell differentiation and cell cycle arrest and inhibits ERK signaling, shifting the phenotype toward efficient neuroblastoma cell death and HDAC8 inhibition affects expression of ALK targets, such as RET (model Fig. 7g).

## Discussion

The use of HDAC inhibitors, which are most commonly utilized as broad-spectrum agents inhibiting the activity of multiple HDAC isotypes, is emerging as an effective cancer treatment strategy [41, 42], and several inhibitors are in phase I-III clinical trials. However, simultaneous inhibition of several HDAC family members confers greater toxicity, resulting in dose-limiting side effects that restrict the anticancer potential of these inhibitors. Using HDAC8-dependent neuroblastoma tumor models, we previously demonstrated that inhibition of a single HDAC isotype is more effective and less toxic than unspecific HDAC inhibition [10]. In comparison to the very sensitive cell line IMR-32, responding to the treatment by cell death, some neuroblastoma cell lines, such as SK-N-BE(2)-C, respond with a differentiation-like phenotype characterized by cell cycle arrest and outgrowth of neurite-like extensions.

### ALK activity and downstream pathways

Here, we identified the ALK pathway as an HDAC8 inhibitor resistance pathway. ALK-activating point mutations predominate in neuroblastoma [43, 44], making it an attractive therapeutic target. ALK mutations, e.g., germline ALK R1275Q, enable the constitutive activation of the kinase domain, which drives tumor cell malignancy and in vivo tumorigenicity [45, 46]. Studies in neuroblastoma have revealed that R1275Q and wild-type ALK-amplified cell lines are highly sensitive to crizotinib [44, 47, 48], whereas cell lines harboring the ALK F1174L mutation are less so, but still more responsive to treatment than non-amplified, wild-type ALK cell lines [44, 47]. The F1174L mutation bears strong oncogenic capacity and correlates with *MYCN* amplification, potentiating the oncogenic activity of MYCN in neuroblastoma, and is linked to acquired resistance to crizotinib [30, 49]. Moreover, mutant *ALK* drives the expression of the tyrosine kinase RET, which is a sympathetic neuronal marker of the cholinergic lineage, but also a proto-oncogene and promising drug target in the context of aberrant ALK activation [40]. In line with the results of Lambertz et al. ALK inhibition alone did not affect RET expression in the ALK wildtype cells, whereas the combination of ALK and HDAC8 inhibitors decreased *RET* expression in our study. Inhibition of HDAC8 counteracted the downregulation of MAPK negative feedback regulator *SPRY4*, which might explain the enhanced inhibitory effect of combination treatment on MAPK/ERK signaling.

ALK aberrations are found in 14% (10% mutations, 4% amplification) of high-risk neuroblastoma patients, and are biomarkers of poor outcome [50]. In neuroblastoma, ALK is almost ubiquitously expressed on the cell surface and expression is restricted to tumor cells [51, 52]. Duijkers et al. [53] demonstrated that, while neuroblastoma cells often express ALK at high levels, the expression in mutated cell lines is even higher with superior responses to inhibition. In our study, all investigated cell lines responded to the combination treatment of crizotinib with the HDAC8 inhibitor, suggesting a sensitization effect independent of ALK mutation or ALK amplification. Of note, the combination treatment of crizotinib with the HDAC8 inhibitor affected neither untransformed cells, nor other embryonic cancer cell models. Our results support at least two mechanisms of action at the level of RTKs, inhibition of the MAPK/ERK pathway and/or induction of the PI3K/AKT/mTOR axis. Although HDACs directly regulate gene transcription, e.g., via histone deacetylation and as interaction partners in corepressor complexes, so far no HDAC8-containing corepressor complex has been identified [54]. In contrast, many non-histone substrates have been described, such as SMC3 [55], ARID1A [56], and nuclear receptor ERRalpha [57]. The list of potential HDAC8 substrates increased massively after the description of the human acetylome by Choudhary et al. [58]. Hence, future studies will elucidate whether indirect effects mediated by one of the potential HDAC8 substrates alter PI3K signaling in neuroblastoma. Several studies have described a role of factor (e.g., IGF, NGF)-mediated activation of the PI3K/AKT/mTOR/S6K signaling pathway in peripheral nerve outgrowth and branching [59–64]. It is conceivable that activation of this pathway mediates the HDAC8 inhibitor-induced priming of neuroblastoma cells for neurite extension and branching [10].

## Conclusion

We have shown that HDAC8 and ALK pathways converge at downstream nodes, and that simultaneous inhibition of HDAC8 and the RTK-ERK pathway leads to efficient cell death of neuroblastoma cells (model Fig. 7g). Altogether, our results provide a solid rationale for the combination treatment of HDAC inhibitors with TKIs, such as crizotinib, which shifts cell cycle arrest and the differentiation phenotype toward effective tumor cell death.

## Methods

### Cell culture, transfections, and reagents

All cell lines were grown under standard conditions as described previously [65]. Human neuroblastoma cell lines SK-N-BE(2)-C (European Collection of Authenticated Cell Cultures (ECACC), Salisbury, UK), IMR-32 (DSMZ, Braunschweig, Germany), Kelly (DSMZ), NB-1 (#RCB1953, RIKEN cell bank, Japan), LAN-5 (generously provided by L. Savelyeva, laboratory of F. Westermann, DKFZ, Germany) and tetracycline-inducible *shMYCN* IMR5/75 [66] (generously provided by the laboratory of F. Westermann), were grown under standard conditions in Dulbecco’s modified Eagle’s medium (DMEM) with l-glutamine, 4.5 g/l glucose (Lonza, Basel, Switzerland) and 1% non-essential amino acids (NEAA) (Invitrogen, Darmstadt, Germany) or in RPMI1640 with l-glutamine (Lonza) and 1% NEAA. All media were supplemented with 10% fetal bovine serum (FBS) (Sigma, Munich, Germany). The embryonal rhabdomyosarcoma cell line RD (kindly provided by S. Fulda, University of Frankfurt, Germany) was grown in DMEM plus GlutaMAX^TM^-I supplemented with 10% FBS. Non-transformed, proliferatively active primary human foreskin fibroblasts from an infant donor (VH7) were a gift from Petra Boukamp, German Cancer Research Center (DKFZ), Heidelberg, Germany. Fibroblasts were maintained in DMEM/Ham’s F12 (Invitrogen) supplemented with 10% FBS and 1% NEAA. All cell lines were genotyped and routinely tested for mycoplasma contamination. The ALK status of neuroblastoma cell lines was confirmed by sequencing (SK-N-BE(2)-C, Kelly) and compared to the literature [53, 67]: SK-N-BE(2)-C: wild type. IMR-32: partial ALK amplification [67]. NB-1: ALK-amplified. LAN-5: ALK R1275Q mutated. Kelly: ALK F1174L mutated. Transient transfections were performed as described previously [9]. The final RNAi concentration was 20 nM for all experiments. All RNAi-IDs used in the screen are listed in Supplementary Table 1 (Dharmacon). In addition, HDAC8 (siRNA1, ID 120597 and siRNA2, ID 120599, Ambion, Huntingdon, UK), ALK (siRNA3, D-003103-07 and siRNA4, D-003103-09 Dharmacon, GE Healthcare) siRNAs and the corresponding negative control siRNA (Silencer Negative Control 1, Silencer Negative Control 5; Ambion and RLuc Duplex (Dharmacon) were used.

### Reagents

HDAC8-selective inhibitors Cpd2 [13] (stock concentration 250 mM) and PCI-34051 [14] (stock 20 mM; Pharmacyclics Inc., Sunnyvale, CA, USA) were dissolved in DMSO. Crizotinib (PF-02341066, stock 10 mM in DMSO, Selleckchem, Houston, USA) and LDK378 (stock 10 mM in DMSO, Selleckchem, Houston, USA) were used as ALK inhibitors. Erlotinib (stock 20 mM; Focus Biomolecules), rapamycin (stock 1 mM; Sigma), LY294002 (stock 10 mM, Cayman) and 20a (Prof. Sippl; University of Halle, Germany) were dissolved in DMSO. EGF stock at 25 µg/ml (PromoKine, Heidelberg, Germany) was also used.

### Kinome-wide siRNA screen

The RNAi screen was performed in duplicate with siRNA-coated 384-well plates using a pooled siRNA library (Dharmacon SMART pool library; Thermo Scientific), where each kinase gene was targeted by a pool of four single siRNAs (Supplementary Table 5). The transfection mix for a single well contained 5 μl siRNApool, 0.05 μl Dharmafect and 14.95 μl RPMI. SK-N-BE(2)-C cells (800 cells/well) were seeded on top, and the final siRNA concentration was 25 nM. Plates were incubated for 24 h. Plates were then treated for 96 h with either HDAC8 inhibitors or solvent (DMSO) control. CTG assays (Promega) were performed to measure cell viability. Medium was removed with a 24-channel comb, and 20 μl of 1:4 diluted CTG reagent was added for 20 min. Luminescence was measured with a Mithras LB940 plate reader (Berthold Technologies). Data were analyzed with web-cellHTS2 [68]. Comparisons of the duplicate experiments revealed high reproducibility of the screen (Supplementary Figure 1A). Positive controls targeting COPB2 (#1), UBC (#2) and PLK1 (#3) and the negative control (Renilla Luciferase) siRNAs were used on each plate to monitor and compare the screen performance of all 18 plates (Supplementary Figure 1B). Mean *z*-factor (all plates): 0.34. Plate-wise *z*-factors (negative control: untreated; positive control: SAHA): 83_1 (Cpd2): 0.54; 83_2 (Cpd2): 0.50; 83_3 (Cpd2): 0.41; 84_1 (Cpd2): 0.19; 84_2 (Cpd2): 0.30; 84_3 (Cpd2): 0.19; 85_1 (PCI-34051): 0.34; 85_2 (PCI-34051): 0.33; 85_3 (PCI-34051): 0.44; 86_1 (PCI-34051): 0.35; 86_2 (PCI-34051): 0.33; 86_3 (PCI-34051): 0.37; 87_1 (DMSO): 0.30; 87_2 (DMSO): 0.27; 87_3 (DMSO): 0.26; 88_1 (DMSO): 0.24; 88_2 (DMSO): 0.37; 88_3 (DMSO): 0.41. Supplementary Figure 1C displays the effect of all non-transfected control treatments, representing relative luminescence units compared with untreated cells, HDAC8 inhibitor (#1 and #2)-treated cells and solvent-treated non-transfected cells. To identify hits that significantly increase (“lethal hits”) or decrease (“rescue hits”) HDAC8i-mediated toxicity, we performed normalization for each treatment. The calculated treatment factor (*t*-factor) was 1.930 +/− 0.07 for HDAC8i #1, 3.789 +/− 0.25 for HDAC8i #2 and 0.959 +/− 0.04 for DMSO. Supplementary Figure 1D displays the data after normalization with the respective factor. A hit was defined as the *t*-factor HDAC8i-corrected t-factor minus the DMSO-corrected *t*-factor >60,000 RLU (=rescue hit) or <−60,000 RLU (=lethality hit). As an additional control, we performed a re-screen with selected hits targeted by single siRNAs in 384-well plates and also validated selected single siRNAs in 96-well plates.

### Cell counting, cell viability, cell death, and colony assay

Cells were collected, pooled with the corresponding supernatant, centrifuged and resuspended in 1.5 ml of cell culture media. Cell counts as well as cell viability were measured by automated trypan blue staining with a Vi-Cell XR Cell Viability Analyzer from Beckman Coulter (Krefeld, Germany). A caspase-3-like protease activity assay was performed as described previously [9]. Apoptosis quantification of propidium iodide-stained and ethanol-fixed cells by flow cytometry was performed as described previously [69]. Colony assay: In six-well plates, 500 cells were seeded and treated for 18–24 h after seeding, as indicated. After 96 h of treatment, the medium was changed to fresh untreated medium allowing the formation of colonies. After a minimum of another seven days, viable colonies were stained with crystal violet (1% in 70% ethanol). For quantification, the plates were scanned, and colonies were counted in 16-bit binary pictures with the ITCN plugin for ImageJ software (U. S. National Institutes of Health, Bethesda, MD, USA; http://imagej.nih.gov/ij/).

### Western blot analysis

Total cell lysates were obtained with NP-40 lysis buffer (150 mM sodium chloride, 1.0% NP-40, 50 mM Tris, pH 8.0), supplemented with protease inhibitor cocktail (cOmplete mini, Roche) and phosphatase inhibitors (PhosSTOP, Roche). Protein samples were denatured with 2-mercaptoethanol at 95 °C for 5 min. The following antibodies were used for detection: anti-pALK Y1604 (Cell Signaling Technology), anti-ALK (Cell Signaling Technology), anti-pSTAT3Y705 (Cell Signaling Technology), anti-STAT3 (Cell Signaling Technology), anti-pERK1/2 (Cell Signaling Technology), anti-ERK1/2 (Cell Signaling Technology), anti-pAkt Y473 (Cell Signaling Technology), anti-AKT, anti-human PARP (Santa Cruz Biotechnology), anti-HDAC8 (H-145;polyclonal; Santa Cruz, Santa Cruz, CA, USA), anti-p-mTOR (Ser2448; Upstate), anti-p-S6K1 (Thr412; Upstate), anti-MET (Cell Signaling Technology), anti-MYCN (Santa Cruz), anti-ac-SMC3 (provided by Prof. K Shirahige, University of Tokyo, Tokyo, Japan) [55], anti-HSC70 (Santa Cruz), anti-β-actin (clone AC-15; Sigma), anti-actinin (H-2; Santa Cruz) and anti-GAPDH (clone 6C5; Merck).

### Real-time, reverse-transcription polymerase chain reaction

Real-time polymerase chain reaction (PCR) was performed as described previously [9]. Data were normalized against the housekeeping genes *SDHA* and *HPRT* [70] and set in relation to a negative control. The following specific primer pairs were used: *CDKN1A* (p21WAF1/CIP1) (forward: 5′-TGG AGA CTC TCA GGG TCG AAA-3′, reverse: 5′-GGC GTT TGG AGT GGT AGA AAT C-3′), *HPRT* (forward: 5′-TGA CAC TGG CAA AAC AAT GCA-3′, reverse: 5′-GGT CCT TTT CAC CAG CAA GCT-3′), *NTRK1* (forward: 5′-CAG CCG GCA CCG TCT CT-3′, reverse: 5′-TCC AGG AAC TCA GTG AAG ATG AAG-3′), *SDHA* (forward: 5′-TGGGAACAAGAGGGCATCTG-3′, reverse: 5′-CCACCACTGCATCAAATTCATG-3′).

### Immunofluorescence and 4′,6-diamidino-2-phenylindole staining

SK-N-BE(2)-C cells (3 × 10^4^) were grown on 8-well chambers (ibidi). Six days after treatment with compounds, cells were fixed for 15 min in 2% (w/v) paraformaldehyde and permeabilized for 15 min with 0.1% (v/v) Triton X-100 in PBS. After washing thrice with PBS, cells were blocked [PBS-Triton with 10% goat serum and 0.25% (w/v) BSA] for 1 h at room temperature, incubated overnight with anti-NEF-M antibody (polyclonal rabbit, Millipore; 1:500) at 4 °C, washed with PBS, and incubated with Cy3-labeled goat anti-rabbit antibody (Dianova; 1:200) for 3 h at room temperature. Nuclei were co-stained with 250 ng/mL 4′,6-diamidino-2-phenylindole (DAPI) solution and analyzed with an Olympus CKX41 microscope and ColorView I FW camera.

### Statistical analysis

All cell culture experiments were performed in duplicate or triplicate, and each experiment was repeated at least three times. A two-tailed *t*-test, unpaired or paired where appropriate, was performed using GraphPad Prism Version 5.00 (GraphPad Software) to compare treatment groups. *p*-values of less than 0.05 were considered significant. The relationship between *HDAC8* and *ALK* expression was determined using the Pearson product-moment correlation, where the *p*-value reflects the result of a *t*-test with the null hypothesis that the correlation between the variables is equal to zero. Kaplan–Meier curves were compared using a log-rank test, where the *p*-value reflects whether the difference between the survival curves is significant. The software program R (R version 3.2.2, 2015; The R Foundation for Statistical Computing) was used to calculate correlation coefficients, identify differentially expressed genes, generate survival curves and perform associated calculations and Cox regression analyses.

### Gene expression analysis

#### Web-based

R2 (R2: microarray analysis and visualization platform; http://r2.amc.nl) was used to investigate HDAC8 and ALK expression in publicly available cohorts of primary neuroblastoma patients. For the Academic Medical Center (AMC) cohort (Gene Expression Omnibus (GEO) database accession no. GSE16476), expression of HDAC8 was detected using the probeset 223345_at, and ALK expression was detected using the probeset 208211_s_at. Patient characteristics were published previously [26]. Expression data for the German Neuroblastoma cohort (GEO database accession no. GSE45547) was obtained from R2 using the probeset Ukv4_A_24_P254965 to detect HDAC8 expression and the probeset Ukv4_A_23_P324304 to detect ALK expression. Patient characteristics were published previously [27]. Current survival data for the German Neuroblastoma cohort were provided by M. Fischer (University of Cologne, Germany).

Gene ontology enrichment analysis was performed with GOrilla (http://cbl-gorilla.cs.technion.ac.il/), pathway analysis for the detection of significantly enriched signaling pathways was performed with REACTOME (http://www.reactome.org/), and a protein class analysis was performed based on the PANTHER classification system [15].

#### Own data

Total RNA was isolated from three independent neuroblastoma cell cultures samples, using the RNeasy MiniKit (Qiagen). For microarray analysis, 1 µg RNA per sample was used. Gene expression analysis was performed at the house-internal Genomics and Proteomics Core Facility using human whole genome HT-12 v4 BeadChips®. Normalization of the raw intensity data was performed by the microarray unit of the DKFZ Genomics and Proteomics Core Facility with Illumina BeadStudio Data Analysis Software version v4_r2. GEO number: GSE110817.  Differentially regulated genes in SK-N-BE(2)-C cells after six days of treatment with PCI-30451 4 µM compared with solvent control were identified using the *topTable* function in the limma package in R [71]. A list of the most differentially expressed genes was created using the following fold change cutoffs: 1.5-fold or more for upregulation and 0.67-fold or less for downregulation.

### Zebrafish lines

Care and breeding of zebrafish were done under standardized conditions. Zebrafish wild-type TE line was raised at 28 °C. Embryos used for tumor injections were maintained in E3 buffer supplemented with 0.2 mM 1-phenyl-2-thiourea (PTU, Sigma).

### Cell preparation and xenotransplantation

SK-N-BE(2)-C cells were cultured to 70–80% confluence, then washed once with PBS (Lonza, Basel, Switzerland), trypsinized (Gibco), counted and resuspended in phenol red-free Roswell Park Memorial Institute medium (RPMI, Gibco). Tumor cells were labeled by incubation with CellTracker CM-DiI (Thermo Fisher Scientific, Waltham, MA, USA) for 5 min at 37 °C, and then for an additional 15 min at 4 °C. To minimize cell clumping, DNase I (250 Kunitz units/ml, Sigma) was added to the cell suspension. Following the incubation, cells were washed twice with 10% FCS RPMI, twice with serum-free RPMI and resuspended in serum-free RPMI to a final concentration of 1.0 × 10^8^ cell/ml. Before implantation, zebrafish were anesthetized with tricaine (0.02%, Sigma) and embedded in a lateral position in 1.0% low gelling temperature agarose (Sigma). Between 150 and 250 CM-DiI-labeled tumor cells were injected into the yolk sac of each zebrafish larva using FemtoJet express microinjector (Eppendorf, Hamburg, Germany) and glass microinjection needles (Science Products, Hofheim, Germany). Larvae were transferred to 34 °C 1 h after tumor cell injection.

### Drug treatment and efficiency evaluation

Tumor xenografts were evaluated by fluorescence microscopy (Olympus, Hamburg, Germany) 2 h post implantation. Only larvae with red fluorescence at the injection site were used for drug testing. Selected embryos were transferred to 48-well uncoated plates (Corning) and incubated in freshly prepared E3 medium containing drugs or solvent. The medium was replaced daily. Tumor growth was evaluated by confocal microscopy before drug exposure as well as 48 h post treatment. For imaging, fish were anesthetized with tricaine (0.02%, Sigma) and embedded in a lateral position in 1.0% low gelling temperature agarose (Sigma) in chambered coverslips (ibidi, Martinsried, Germany). Images of living larvae were obtained using a Zeiss LSM 710 confocal microscope (Zeiss, Oberkochen, Germany) and ZEN software (Zeiss). Tumor progression was evaluated using Fiji software and a semi-automated macro. Zebrafish embryos were treated with a ten-fold higher concentration of crizotinib (8 µM) and 20a (100 µM) as substances are applied to the surrounding water and, as the estimated extent of compound absorption by the zebrafish larvae is one-tenth to one-twentieth of the cell culture treatment concentration [28, 72].

## Electronic supplementary material

Supplementary data

Supplemental Table 1

Supplemental Table 2

Supplemental Table 3

Supplemental Table 4

Supplemental Table 5

Supplemental Figure 1

Supplemental Figure 2

Supplemental Figure 3

Supplemental Figure 4

Supplemental Figure 5
