# Supplementary material for: A kinome-wide RNAi screen identifies ALK as a target to sensitize neuroblastoma cells for HDAC8-inhibitor treatment
Source: Cell Death Differ. 2018 Mar 7;25(12):2053–70. doi: 10.1038/s41418-018-0080-0 (PMC6261943; doi:10.1038/s41418-018-0080-0)
Supplement: Supplementary file 1 — Supplementary data [file 41418_2018_80_MOESM1_ESM.docx]

**Supplementary data**

**A kinome-wide RNAi screen identifies ALK as a target to sensitize neuroblastoma cells for HDAC8-inhibitor treatment**

Jing Shen^1^, Sara Najafi^1,2^, Sina Stäble^1^, Johannes Fabian^1, 3^, Emily Koeneke^1,2^, Fiona R. Kolbinger^1,2^, Jagoda Wrobel^1,2^, Benjamin Meder^4^, Martin Distel^5^, Tino Heimburg^6^, Wolfgang Sippl^6^, Manfred Jung^7^, Heike Peterziel^1,2^, Dominique Kranz^8^, Michael Boutros^8^, Frank Westermann^9^, Olaf Witt^1,2,10^, Ina Oehme^1,2,^ *

^1^Clinical Cooperation Unit Pediatric Oncology, German Cancer Research Center (DKFZ)

^2^Preclinical Program, Hopp Children’s Cancer Center at NCT Heidelberg (KiTZ)

^3^current address: Phenex Pharmaceuticals AG, Heidelberg, Germany

^4^Institute for Cardiomyopathies Heidelberg, Heidelberg University, Germany

^5^Innovative Cancer Models, St. Anna Children’s Cancer Research Institute, Vienna, Austria

^6^Institute of Pharmacy, Martin-Luther University of Halle-Wittenberg, 06120 Halle/Saale, Germany

^7^Institute of Pharmaceutical Sciences, University of Freiburg, 79104 Freiburg, Germany

^8^Division of Signaling and Functional Genomics, German Cancer Research Center and Heidelberg University, Department for Cell and Molecular Biology, Medical Faculty Mannheim, Heidelberg, Germany

^9^Research Group Neuroblastoma Genomics, German Cancer Research Center

^10^Department of Pediatric Oncology, Hematology and Immunology, University of Heidelberg Medical Center

* corresponding author: i.oehme@dkfz.de

Supplemental Figure 1

**Kinome-wide RNAi screen for the identification of HDAC8 inhibitor sensitizing co-treatments. (A)** Scatterplots displaying the relative luminescence (10^5^) between two replicate plates, measured with Cell Titer Glo, r_s_: Spearman rank correlation. HDAC8i: HDAC8 inhibitor (#1: 40 µM Cpd2, #2: 4 µM PCI-34051).  **(B)** Bar diagram displaying normalized RLU relative to untreated cells for positive (PC) and negative (NC) controls measured via Cell Titer Glo. NC: Renilla luciferase (RL) siRNA, PC#1: COPB2 siRNA, PC#2: UBC siRNA, PC#3: PLK1 siRNA. **(C)** Bar diagram displaying RLU for untreated (UN), HDAC8 inhibitor (HD8) and solvent (DMSO) treated SK-N-BE(2)-C cells, measured via Cell Titer Glo. **(D)** Bar diagram displaying normalized and treatment factor corrected RLU for HDAC8 inhibitor (HD8) and DMSO (solvent) treated SK-N-BE(2)-C cells, measured via Cell Titer Glo. **(E)** Gene Ontology enrichment result for the “rescue hit” list obtained with the Gene Ontology enRIchment anaLysis and visuaLizAtion tool (GOrilla; http://cbl-gorilla.cs.technion.ac.il/).

Supplemental Figure 2

***In vivo* evaluation of the dual targeting of ALK and HDAC8 (A)** Change of tumor volume from day 1 to day 3 post-implantation of SK-N-BE(2)-C neuroblastoma xenografts in zebrafish treated with either solvent (DMSO, grey), HDAC8 inhibitor 20a (100 µM, green), crizotinib (8 µM, blue) or the combination of both (orange). Treatment started 1 day after implantation of fluorescently-labeled SK-N-BE(2)-C cells into the yolk sac of the embryos (black arrow). Data are represented for each individual xenograft. **(B)** Determination of MTD (maximum tolerated dose). Two zebrafish larvae per group were treated for 72 hours with compounds in increasing concentrations and monitored for toxicity (defined as undeveloped or dead larvae) each day. Red cross: signs of toxicity for both larvae; green check mark: both larvae are healthy and developing.

Supplemental Figure 3

**Dual targeting of ALK and HDAC8 induces apoptosis. (A)** Caspase-3-like (DEVDase) activity was assessed using a fluorometric assay with activity (slope/min) measured relative to untreated cells. Neuroblastoma cells were treated with crizotinib (Kelly, 0.8 µM; NB-1 0.05 µM) alone or in combination with PCI-34051 (6 µM) and then monitored 96 h after treatment for caspase activity. **(B)** Kelly cells were treated as indicated and 96 h later, the cells and supernatants were collected for the detection of apoptotic cell death by propidium iodide (PI) staining of ethanol-fixed cells. **(C)** Kelly cells were treated with crizotinib (0.8 µM), alone or in combination with PCI-34051 (6 µM), 48 h later Caspase inhibitor zVAD.fmk (50 µM) was added and cells were monitored 96 h after the first treatment for cell death using trypan blue staining (dead cells: trypan blue positive cells). **(D)** Histograms for the detection of apoptotic cell death by propidium iodide (PI, FL2) staining of ethanol-fixed cells. Kelly cells were treated with crizotinib (0.8 µM), alone or in combination with PCI-34051 (6 µM). After 96 h, the cells and supernatants were collected. **(E)** Kelly cells were treated with LDK378 (0.8 µM), alone or in combination with PCI-34051 (6 µM) and 96 h later, the cells and supernatants were collected for the detection of apoptotic cell death by propidium iodide (PI) staining of ethanol-fixed cells. **(F)** Kelly cells were treated with crizotinib (0.8 µM), alone or in combination with 20a (10 µM) and cells were monitored 96 h after treatment for cell death using trypan blue staining (dead cells: trypan blue positive cells). **(G)** LAN-5 cells were treated with crizotinib (0.6 µM), alone or in combination with PCI-34051 (3 µM) and viable cell number was determined 6 d after treatment using trypan blue staining (viable cells: trypan blue negative cells). The representative microscopic pictures display a more differentiated phenotype after PCI-34051 treatment of LAN-5 cells and a shift from differentiation to more floating cells after the combination treatment with PCI-34051 and crizotinib. **(A)-(C), (E)-(G)** Means from at least three independent experiments are shown, and error bars represent SEM. **P* < 0.05; ***P* < 0.01; ****P* < 0.001.

Supplemental Figure 4

**Treatment of neuroblastoma cells with crizotinib inhibits ALK activity**

Detection of ALK protein and phosphorylation (Y1604) levels in SK-N-BE(2)-C and NB-1 cells treated with crizotinib (SK-N-BE(2)-C: 0.6 µM; NB-1: 0.05 µM) alone or in combination with PCI-34051 (6 µM). Numbers indicate pALK levels normalized to ALK expression.

Supplemental Figure 5

**The PI3K/AKT/mTOR pathway specifically counteracts HDAC8 inhibitor mediated effects**

**(A)** SK-N-BE(2)-C cells were treated with BEZ235 (10 nM) alone or in combination with PCI-34051 (6 µM), and RNA was isolated 72 h after treatment for real-time PCR analysis of *CDKN1* (*p21*) expression. **(B)** Kelly cells were treated with LY294002 (10 µM) alone or in combination with PCI-34051 (6 µM), and RNA was isolated 72 h after treatment for real-time PCR analysis of *CDKN1* (p21) expression. **(C)** Detection of p21 protein levels in Kelly cells treated for 96 h with LY294002 (10 µM) alone or in combination with PCI-34051 (4 µM). Numbers indicate p21 levels normalized to β-actin expression. **(D)** Immunofluorescent pictures show neurofilament (red; NEF-M) and DAPI (blue) staining of SK-N-BE(2)-C cells 6 days after treatment with 6 µM PCI-34051, 100 nM rapamycin (100 nM) or the combination of both (scale bar=100 *μ*m). **(E)** SK-N-BE(2)-C cells were treated with LY294002 (10 µM) alone or in combination with entinostat (0.5 µM) and then monitored 96 h after treatment for viable cell number using trypan blue staining. Relative cell number: normalized to solvent (DMSO) treated cells. **(F)** SK-N-BE(2)-C cells were treated with rapamycin (100 nM) alone or in combination with entinostat (0.5 µM) and then monitored 96 h after treatment for viable cell number using trypan blue staining. Relative cell number: normalized to solvent (DMSO) treated cells. **(G)** Venn diagram depicting the overlap of differentially expressed genes in SK-N-BE(2)-C cells after six days of PCI-34051 (4 µM) treatment and a published ALK signature of differentially expressed genes in neuroblastoma cells following ALK inhibitor treatment or ALK knockdown (Lambertz *et al.*, Clin Cancer Res. 2015 Jul 15;21(14):3327-39). **(A)-(B),** **(E)-(F)** Means from at least three independent experiments are shown, and error bars represent SEM. ****P* < 0.001.
