# Supplementary material for: A kinome-wide RNAi screen identifies ALK as a target to sensitize neuroblastoma cells for HDAC8-inhibitor treatment
Source: Cell Death Differ. 2018 Mar 7;25(12):2053–70. doi: 10.1038/s41418-018-0080-0 (PMC6261943; doi:10.1038/s41418-018-0080-0)
Supplement: Supplementary file 2 — Supplemental Table 1 [file 41418_2018_80_MOESM2_ESM.docx]

**Supplementary Table 1:** list of all lethality and rescue hits of the kinome-wide RNAi screen

Data is normalized for each treatment. The calculated treatment factor (t-factor) for HDAC8i #1 equals 1.930 +/- 0.07, for HDAC8i #2 equals 3.789 +/-0.25 and for DMSO equals 0.959 +/-0.04. A hit is defined by mean HDAC8i minus mean DMSO > 60,000 or < -60,000. Difference has to be in the same direction for both inhibitors.

Mean untreated: 239858 (RLU). Deviation of 25%: 60,000 (= cut-off).

| **siRNA** | \| **Cpd2 (HDAC8i #1)** \|  \| \| --- \| --- \|   (mean, normalized,  t-factor corrected) | \| **PCI-34051 (HDAC8i #2)** \|  \| \| --- \| --- \|   (mean, normalized,  t-factor corrected) | \| **DMSO** \|  \| \| --- \| --- \|   (mean, normalized,  t-factor corrected) | **difference**  (HDAC8i minus DMSO) | **effect** | **plate** | **well** |
| --- | --- | --- | --- | --- | --- | --- | --- | --- | --- | --- | --- | --- | --- |
| RAPGEF4 | 77851 | 45735 | 170335 | < -60,000 | lethal | 1 | A11 |
| BDKRB2 | 150319 | 157529 | 275484 | < -60,000 | lethal | 1 | A14 |
| CHEK2 | 160265 | 166411 | 287965 | < -60,000 | lethal | 1 | A15 |
| CHKB | 115568 | 132416 | 212344 | < -60,000 | lethal | 1 | A19 |
| ERBB2 | 162875 | 86473 | 233195 | < -60,000 | lethal | 1 | B06 |
| FASTK | 209196 | 151151 | 271982 | < -60,000 | lethal | 1 | B18 |
| CSNK1D | 221219 | 335291 | 143718 | > 60,000 | rescue | 1 | B23 |
| BTK | 56795 | 120009 | 274688 | < -60,000 | lethal | 1 | C16 |
| CLK3 | 85551 | 66226 | 171511 | < -60,000 | lethal | 1 | E05 |
| CAMK2B | 163983 | 197699 | 98932 | > 60,000 | rescue | 1 | G06 |
| ADRA1B | 128957 | 117479 | 276997 | < -60,000 | lethal | 1 | G19 |
| FLT1 | 286945 | 198119 | 393863 | < -60,000 | lethal | 1 | H08 |
| DKFZP434C131 | 357062 | 349192 | 286723 | > 60,000 | rescue | 1 | H13 |
| FRK | 90517 | 98857 | 160578 | < -60,000 | lethal | 1 | H22 |
| AK2 | 112986 | 98138 | 285368 | < -60,000 | lethal | 1 | I11 |
| AK3 | 167351 | 215947 | 405120 | < -60,000 | lethal | 1 | I13 |
| GAK | 73034 | 135355 | 222993 | < -60,000 | lethal | 1 | J10 |
| DUSP1 | 138873 | 134316 | 224711 | < -60,000 | lethal | 1 | J19 |
| AKAP13 | 110078 | 102811 | 280536 | < -60,000 | lethal | 1 | K05 |
| GMFB | 433886 | 405469 | 507037 | < -60,000 | lethal | 1 | L06 |
| GSG2 | 68801 | 67040 | 137053 | < -60,000 | lethal | 1 | L20 |
| ALK | 140128 | 116980 | 201499 | < -60,000 | lethal | 1 | M05 |
| CDK5R2 | 196178 | 159649 | 92742 | > 60,000 | rescue | 1 | M10 |
| CDK8 | 453122 | 618872 | 295735 | > 60,000 | rescue | 1 | M20 |
| CDK9 | 345985 | 290178 | 184286 | > 60,000 | rescue | 1 | M22 |
| GUCY2C | 143627 | 109840 | 217213 | < -60,000 | lethal | 1 | N08 |
| HIPK1 | 148035 | 164069 | 259602 | < -60,000 | lethal | 1 | N22 |
| EPHA2 | 297015 | 205286 | 412169 | < -60,000 | lethal | 1 | N23 |
| ATR | 188102 | 170142 | 328470 | < -60,000 | lethal | 1 | O15 |
| HRI | 30577 | 63289 | 166953 | < -60,000 | lethal | 1 | P16 |
| EPHB2 | 118047 | 109123 | 227622 | < -60,000 | lethal | 1 | P17 |
| EPHB4 | 158627 | 88396 | 232866 | < -60,000 | lethal | 1 | P21 |
| IHPK2 | 99318 | 84808 | 257614 | < -60,000 | lethal | 2 | A11 |
| IKBKB | 100248 | 118320 | 222609 | < -60,000 | lethal | 2 | A17 |
| PKIA | 163904 | 156199 | 224029 | < -60,000 | lethal | 2 | B14 |
| NPR1 | 253788 | 335173 | 189919 | > 60,000 | rescue | 2 | B23 |
| MAPK8IP2 | 156538 | 247212 | 58178 | > 60,000 | rescue | 2 | E12 |
| JIK | 151951 | 239884 | 91896 | > 60,000 | rescue | 2 | E17 |
| PRKAG1 | 158115 | 149048 | 256855 | < -60,000 | lethal | 2 | F12 |
| PRKAG3 | 171603 | 165070 | 97113 | > 60,000 | rescue | 2 | F14 |
| MBIP | 270649 | 408739 | 182198 | > 60,000 | rescue | 2 | G16 |
| MET | 215735 | 311630 | 138044 | > 60,000 | rescue | 2 | G22 |
| PCTK1 | 151213 | 156956 | 90605 | > 60,000 | rescue | 2 | H17 |
| PDGFRA | 281465 | 276116 | 162000 | > 60,000 | rescue | 2 | H23 |
| KIT | 107660 | 146181 | 226234 | < -60,000 | lethal | 2 | I07 |
| PDK4 | 249518 | 304297 | 184584 | > 60,000 | rescue | 2 | J13 |
| PDXK | 381637 | 532450 | 293132 | > 60,000 | rescue | 2 | J17 |
| PRPS1L1 | 156646 | 175581 | 243067 | < -60,000 | lethal | 2 | K13 |
| PRKWNK4 | 79873 | 115166 | 311300 | < -60,000 | lethal | 2 | L10 |
| PRKX | 149402 | 144394 | 233804 | < -60,000 | lethal | 2 | L12 |
| PHKA1 | 241337 | 253568 | 146519 | > 60,000 | rescue | 2 | L19 |
| MVD | 213018 | 233177 | 128792 | > 60,000 | rescue | 2 | M12 |
| MVK | 160901 | 195227 | 88793 | > 60,000 | rescue | 2 | M14 |
| MAP2K1IP1 | 201759 | 165724 | 326652 | < -60,000 | lethal | 2 | M15 |
| MYO3B | 357790 | 451576 | 194109 | > 60,000 | rescue | 2 | M22 |
| MAP2K5 | 271739 | 261498 | 339465 | < -60,000 | lethal | 2 | M23 |
| PI4K2B | 207236 | 330702 | 108153 | > 60,000 | rescue | 2 | N07 |
| PIK3CA | 332240 | 207090 | 100531 | > 60,000 | rescue | 2 | N17 |
| PIK3CB | 170231 | 174990 | 88475 | > 60,000 | rescue | 2 | N19 |
| PIK3R1 | 284339 | 326942 | 223990 | > 60,000 | rescue | 2 | N23 |
| NEK6 | 347937 | 410422 | 256911 | > 60,000 | rescue | 2 | O20 |
| NEK7 | 403183 | 386736 | 287633 | > 60,000 | rescue | 2 | O22 |
| PIK3R4 | 240203 | 239502 | 109265 | > 60,000 | rescue | 2 | P09 |
| PIP5K1A | 320273 | 220186 | 155271 | > 60,000 | rescue | 2 | P21 |
| RFP | 162134 | 239332 | 75744 | > 60,000 | rescue | 3 | A10 |
| TLK1 | 96335 | 104744 | 177841 | < -60,000 | lethal | 3 | C17 |
| RYK | 307862 | 251470 | 371218 | < -60,000 | lethal | 3 | E18 |
| TRIO | 186252 | 175515 | 264219 | < -60,000 | lethal | 3 | E23 |
| SGK | 284111 | 300626 | 219134 | > 60,000 | rescue | 3 | G08 |
| TSKS | 153513 | 145752 | 80562 | > 60,000 | rescue | 3 | G09 |
| SGK2 | 126331 | 226905 | 65894 | > 60,000 | rescue | 3 | G10 |
| SGKL | 423094 | 462150 | 230936 | > 60,000 | rescue | 3 | G12 |
| SHC1 | 299376 | 321093 | 144105 | > 60,000 | rescue | 3 | G14 |
| TYRO3 | 139067 | 127460 | 247160 | < -60,000 | lethal | 3 | I05 |
| UGP2 | 186706 | 185720 | 324598 | < -60,000 | lethal | 3 | I09 |
| ULK2 | 195472 | 161537 | 297721 | < -60,000 | lethal | 3 | I13 |
| SPHK1 | 260259 | 129669 | 46357 | > 60,000 | rescue | 3 | I18 |
| URKL1 | 178176 | 193006 | 117595 | > 60,000 | rescue | 3 | I19 |
| SPHK2 | 222131 | 165674 | 286662 | < -60,000 | lethal | 3 | I20 |
| ABI1 | 254398 | 261776 | 159405 | > 60,000 | rescue | 3 | K14 |
| SSTK | 263827 | 391114 | 148409 | > 60,000 | rescue | 3 | K16 |
| PLK4 | 199249 | 195285 | 89521 | > 60,000 | rescue | 3 | M08 |
| STK19 | 230545 | 248905 | 111751 | > 60,000 | rescue | 3 | M10 |
| STK22C | 362601 | 319538 | 166309 | > 60,000 | rescue | 3 | M14 |
