# Supplementary material for: A kinome-wide RNAi screen identifies ALK as a target to sensitize neuroblastoma cells for HDAC8-inhibitor treatment
Source: Cell Death Differ. 2018 Mar 7;25(12):2053–70. doi: 10.1038/s41418-018-0080-0 (PMC6261943; doi:10.1038/s41418-018-0080-0)
Supplement: Supplementary file 3 — Supplemental Table 2 [file 41418_2018_80_MOESM3_ESM.docx]

**Supplementary Table 2:** Neuroblastoma toxic hits

|  |
| --- |

| **name** | \| **siRNA-DMSO** \| \| --- \|   (plate normalized RLU) | **“untreated” minus “siRNA-DMSO” >200000**  (mean untreated w/o siRNA = 239858) | **plate** | **well** |
| --- | --- | --- | --- | --- | --- |
| BCKDK | 34342 | yes | 1 | A10 |
| BLNK | 14067 | Yes | 1 | A18 |
| BMPR1B | 26713 | Yes | 1 | A24 |
| COPB2 | 15522 | Yes | 1 | B11 |
| ERK8 | 30911 | Yes | 1 | B12 |
| CIT | 34257 | Yes | 1 | C07 |
| BRAF | 24269 | Yes | 1 | C10 |
| FGR | 32444 | Yes | 1 | D12 |
| ADCK2 | 25265 | Yes | 1 | G09 |
| DKFZp434C1418 | 14671 | Yes | 1 | H15 |
| FN3KRP | 30018 | Yes | 1 | H16 |
| DKFZP586B1621 | 22654 | Yes | 1 | H17 |
| DKFZP761P0423 | 33822 | Yes | 1 | H19 |
| CD4 | 16957 | Yes | 1 | I16 |
| DMPK | 39849 | Yes | 1 | J11 |
| CDK6 | 30528 | Yes | 1 | M16 |
| EDN2 | 27173 | Yes | 1 | N07 |
| ITGB1BP1 | 18155 | Yes | 1 | P24 |
| ILK | 38917 | Yes | 2 | A23 |
| NME6 | 30786 | Yes | 2 | B19 |
| IRAK3 | 18669 | Yes | 2 | C17 |
| MAPK3 | 34728 | Yes | 2 | C20 |
| PLK1 | 9861 | Yes | 2 | D06 |
| PRKAA2 | 18312 | Yes | 2 | D24 |
| MAST3 | 38105 | Yes | 2 | G05 |
| PANK4 | 29504 | Yes | 2 | H05 |
| PASK | 23998 | Yes | 2 | H11 |
| PRKCH | 39161 | Yes | 2 | H14 |
| PCK2 | 38855 | Yes | 2 | H15 |
| LAK | 36760 | Yes | 2 | I13 |
| PRKDC | 22158 | Yes | 2 | J14 |
| PFKFB1 | 34684 | Yes | 2 | J19 |
| MYLK2 | 29582 | Yes | 2 | M18 |
| MAP2K7 | 38040 | Yes | 2 | O07 |
| COASY | 26678 | Yes | 2 | O08 |
| PTPRJ | 14171 | Yes | 2 | P08 |
| SYK | 21539 | Yes | 3 | A05 |
| RIOK1 | 37496 | Yes | 3 | A14 |
| RIPK2 | 33300 | Yes | 3 | A20 |
| TESK2 | 36665 | Yes | 3 | A21 |
| TEX14 | 19528 | Yes | 3 | A23 |
| RSEL | 29711 | Yes | 3 | A24 |
| RPS6KA2 | 14765 | Yes | 3 | C20 |
| RPS6KA5 | 29339 | Yes | 3 | E06 |
| TRIB2 | 32555 | Yes | 3 | E19 |
| SNRK | 18674 | Yes | 3 | I08 |
| WEE1 | 12557 | Yes | 3 | K07 |
