# Supplementary material for: A kinome-wide RNAi screen identifies ALK as a target to sensitize neuroblastoma cells for HDAC8-inhibitor treatment
Source: Cell Death Differ. 2018 Mar 7;25(12):2053–70. doi: 10.1038/s41418-018-0080-0 (PMC6261943; doi:10.1038/s41418-018-0080-0)
Supplement: Supplementary file 4 — Supplemental Table 3 [file 41418_2018_80_MOESM4_ESM.docx]

**Suppl. Table 3**

Stratified multivariate Cox regression model and hazard ratio

| **Variable** | **Effect** | **Hazard ratio** | ***P*-value** |
| --- | --- | --- | --- |
| *HDAC8* and *ALK* Expression | *HDAC8* and *ALK* both above median vs one or neither | 5.11 | <0.001 |
| Stage | Stage 4  vs Stages 1,2,3 and 4s | 9.38 | <0.001 |
| Expression : Stage | interaction term | 0.3 | <0.01 |

Note: Model was stratified by the variables age (dichotomous: age over 18 months vs younger than 18 months) and *MYCN* amplification (dichotomous: amplified vs non-amplified). These variables failed to meet the proportional hazards assumption.
