# Supplementary figures and images for: A kinome-wide RNAi screen identifies ALK as a target to sensitize neuroblastoma cells for HDAC8-inhibitor treatment
Source: Cell Death Differ. 2018 Mar 7;25(12):2053–70. doi: 10.1038/s41418-018-0080-0 (PMC6261943; doi:10.1038/s41418-018-0080-0)

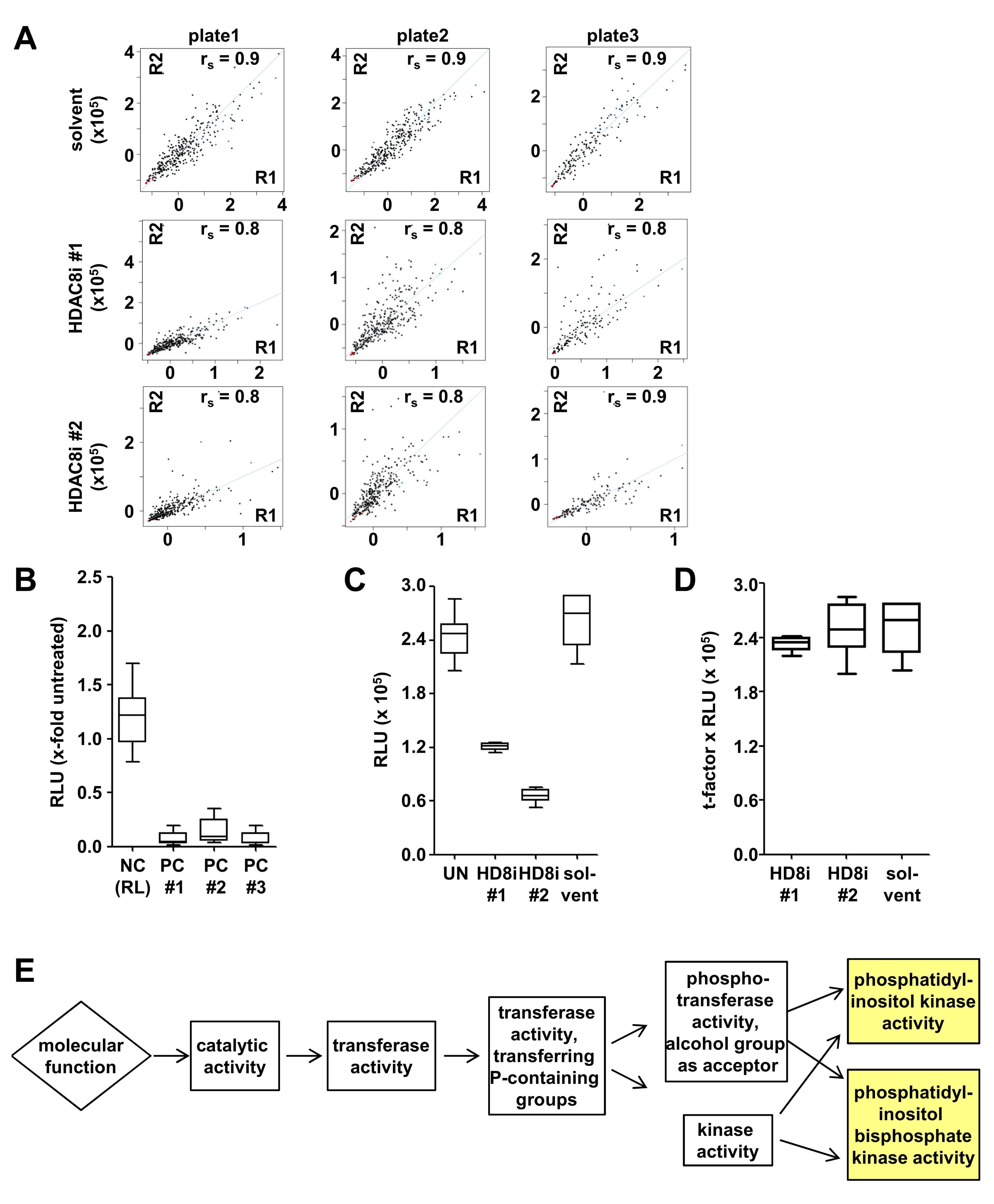

Supplement: Supplementary file 7 — Supplemental Figure 1 [file 41418_2018_80_MOESM7_ESM.tif]

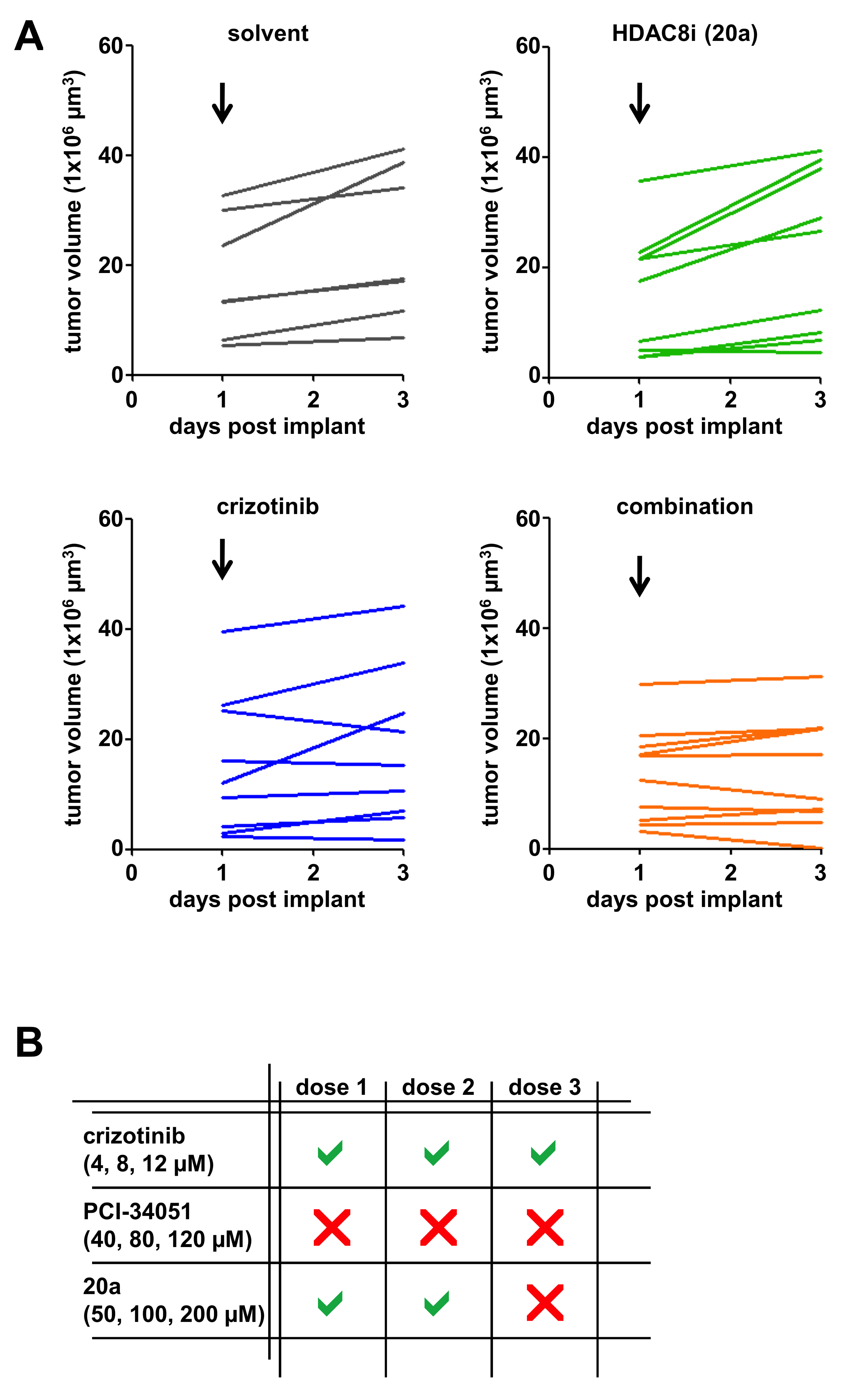

Supplement: Supplementary file 8 — Supplemental Figure 2 [file 41418_2018_80_MOESM8_ESM.tif]

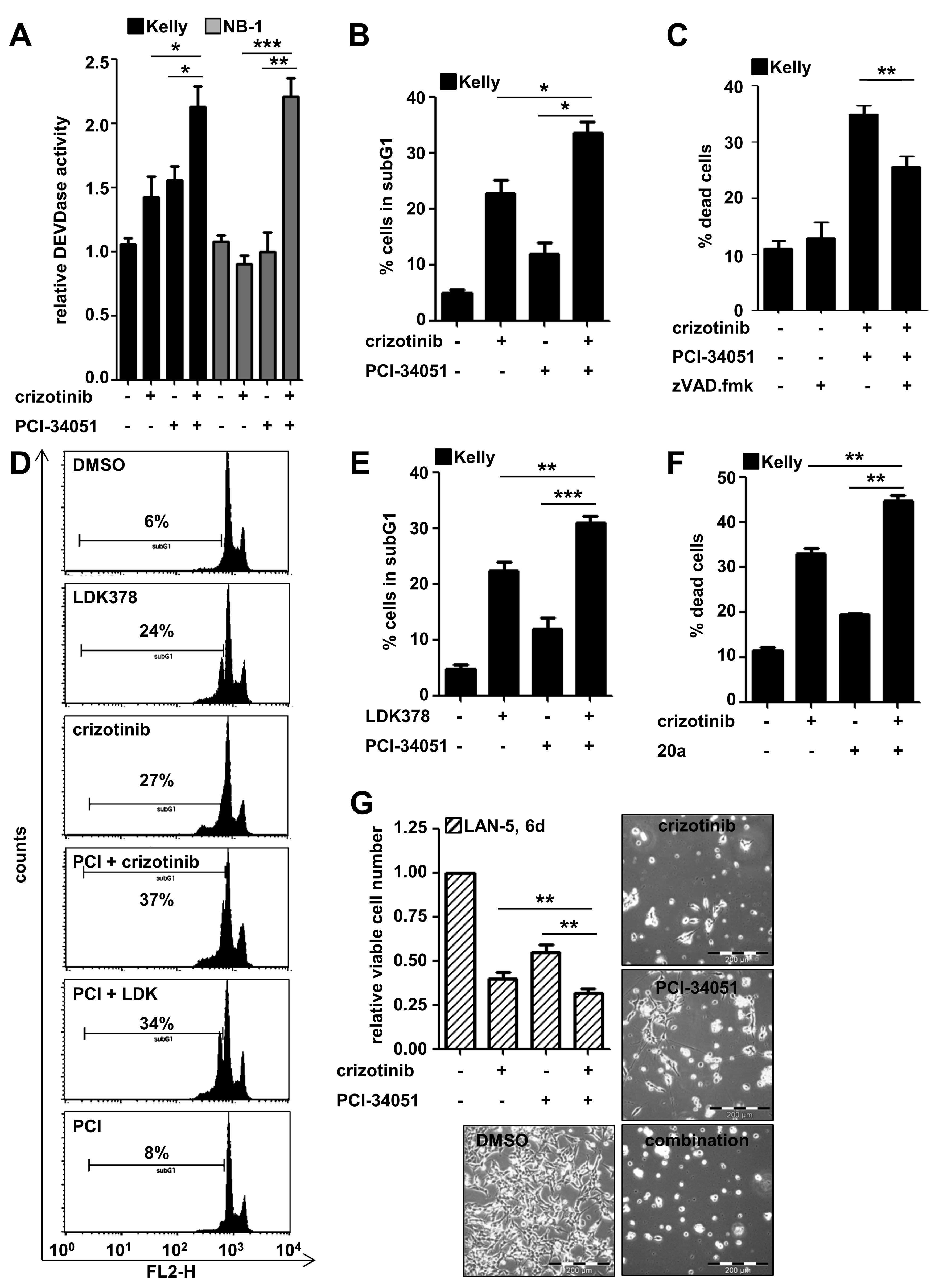

Supplement: Supplementary file 9 — Supplemental Figure 3 [file 41418_2018_80_MOESM9_ESM.tif]

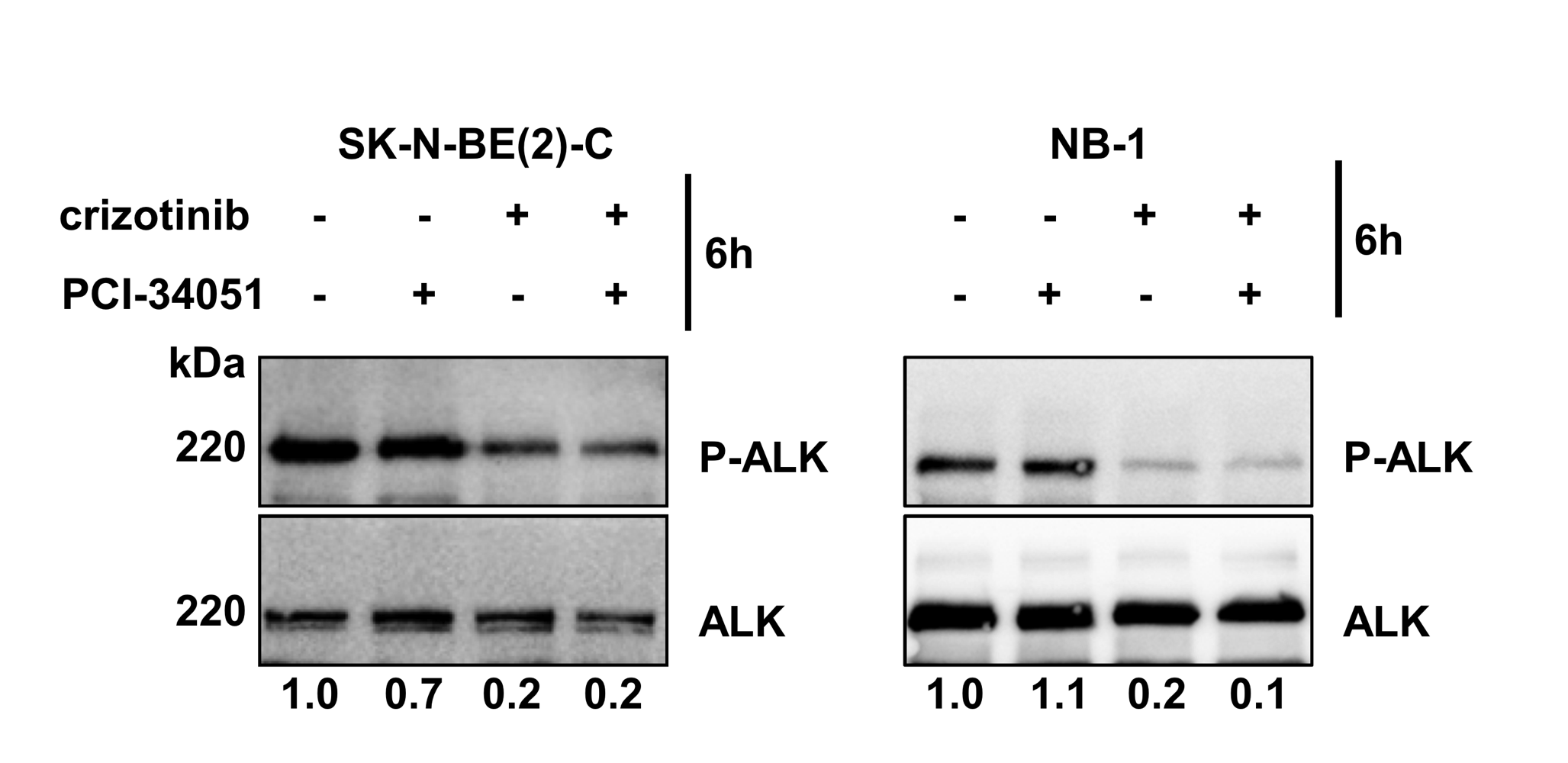

Supplement: Supplementary file 10 — Supplemental Figure 4 [file 41418_2018_80_MOESM10_ESM.tif]

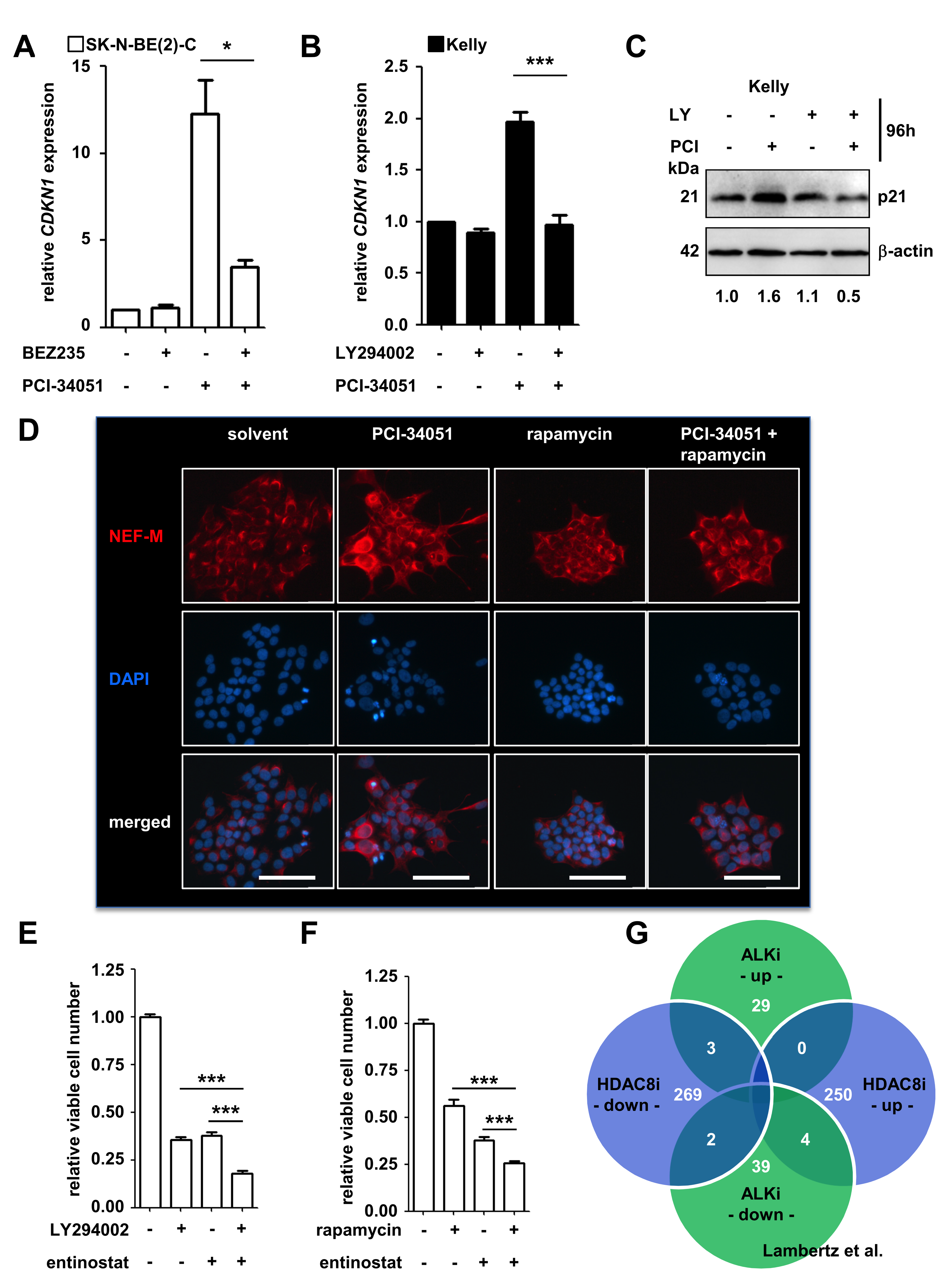

Supplement: Supplementary file 11 — Supplemental Figure 5 [file 41418_2018_80_MOESM11_ESM.tif]
